# Bioinspired Asymmetrical Pleated Textile With Unidirectional Transport Channel for Personal Moisture and Thermal Management

**DOI:** 10.1002/EXP.20240357

**Published:** 2025-06-12

**Authors:** Meitong Ge, Fengxiang Chen, Chaoyu Chen, Honglian Cong, Xin Wang, Zhijia Dong, Pibo Ma

**Affiliations:** ^1^ Engineering Research Center of Knitting Technology Ministry of Education College of Textile Science and Engineering Jiangnan University Wuxi China; ^2^ State Key Laboratory of New Textile Materials and Advanced Processing Technologies/National Local Joint Laboratory for Advanced Textile Processing and Clean Production/Hubei Key Laboratory of Digital Textile Equipment Wuhan Textile University Wuhan P. R. China; ^3^ School of Fashion and Textiles RMIT University Melbourne Australia

**Keywords:** asymmetric structures, directional water transport, liquid management, thermal‐moisture comfort

## Abstract

Asymmetrical pleated textile with unidirectional water transport plays a vital role in maintaining personal moisture and thermal comfort. Inspired by the cactus branch, in this work, an asymmetrical pleated structure textile embedded with a unidirectional water transport channel was proposed by seamless weft knitting technology. This innovative textile with differential capillary effect can swiftly transport water within 1 s, with an accumulative one‐way transport index (AOTI) of 499.57%. This textile also exhibits excellent external water repellency with a stable contact angle exceeding 120°. Most importantly, water repellency, water collection, and directional water transport ability are integrated into one unified system by means of the asymmetrical pleated structure, thereby ensuring both safety and comfort for the wearer. The advanced fabrics meet high transmission indexes and fast transport rates, which are expected to provide a fresh avenue for the development and creation of more efficient and adaptive personal moisture and thermal management fabrics.

## Graphical abstract

Inspired by the cactus branch, this work proposed an asymmetrical pleated structure textile embedded with a unidirectional water transport channel. Water repellency, water collection, and directional water transport ability are assembled into one unified system by means of the asymmetrical pleated structure, which is expected to enable efficient moisture regulation for humans.

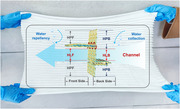

## 1 Introduction

Functional textiles with dynamic moisture management have received much attention for maintaining the individual's comfort. It is widely known that sweat evaporation is a proven method for heat diffusion in the human body, which can reduce the skin temperature and create an appropriate microclimate [1]. However, traditional clothing fabrics tend to become heavy and sticky when saturated with sweat, resulting in an uncomfortable damp and cold feeling [2]. Additionally, these fabrics do not provide adequate protection against the penetration and absorption of external liquids. Therefore, there is a pressing need for the development of next‐generation moisture management fabrics that not only remove and dissipate sweat directionally but also effectively repel and resist external liquids.

Recently, the functional materials with an ability of dynamic liquid management have attracted much attention. These materials can be obtained through physical and chemical modification, such as plasma treatment [3], laser perforation [4], or the combination of yarns [5], in order to create the distinct surface wettability, micro‐/nano‐structure, and geometric shape, achieving asymmetric gradient wettability and differential capillary effect [4, 6]. For example, Dai et al. [4a] proposed a hydrophobic/superhydrophilic Janus polyester/nitrocellulose textile embedded with a conical micropore array with a hydrophilic inner surface, allowing automatically directional water transport because of the capillary force. However, the external side of fabric which developed by the above mentioned methods is usually designed to be hydrophilic. While this hydrophilic property effectively facilitates water transport, it often leads to the overlooked issue of excessive liquid removal. Even worse, those fabrics may suffer from heavy weight because of liquid saturation. Under such saturated conditions, the efficiency of liquid water transfer diminishes, as water is removed more slowly through evaporation compared to direct liquid removal. Consequently, the effect of excessive water absorption hinders the comprehensive performance of Janus fabrics in managing moisture effectively. To overcome the drawbacks, construction of a 3D structure, such as conical spine [7], origami process [8], and hybrid channel [9], has been an effective method for designing functional textiles with effective liquid management capability, inducing directional water transport through anisotropic capillary. However, the current design of fabric was typically designed with a flat structure, resulting in (1) prolonged micro‐wetting on the inner next‐to‐skin side, (2) increased weight and stickiness once the outer layer became saturated, (3) difficulty in repelling external water, and (4) an unsightly appearance of the garment after being wetted with sweat. While the effectiveness of directional liquid transport fabrics has been confirmed in laboratory settings, challenges persist regarding their liquid management capabilities and practical application. Therefore, the development of a new functional textile to achieve efficient sweat transport and prevent outside water permeation is urgently needed.

The animals and plants in nature, such as the back of beetles, the peristome of pitcher plants, and the spines of cacti, spontaneous and directional liquid transport phenomena can be widely found due to the heterogeneous geometry and gradient wettability [4, 10], which inspired scholars a lot. 3D structure is conducive to achieve differential capillary configured on the different sides of materials, generating asymmetric Laplace pressure. For example, the conical fishbone which possesses a fantastic low‐surface‐tension microdroplets steering ability can generate asymmetric Laplace pressure and enable the droplet directional sliding [7]. According to the Laplace equation, the thinner capillary tube is, the stronger water absorption it has, hence the liquid would be easier to achieve automatic ascent and transportation. Moreover, the hydrophobic treatment on the surface can prevent the external liquid incursion, which can be achieved by lower surface energy or the construction of micro‐nano rough surface [11]. For example, the Laplace pressure, created by the wettability gradient and hydrophobic cacti spines with gradient conical structure, enable together continuous and efficient water collection and transport.

**FIGURE 1:**
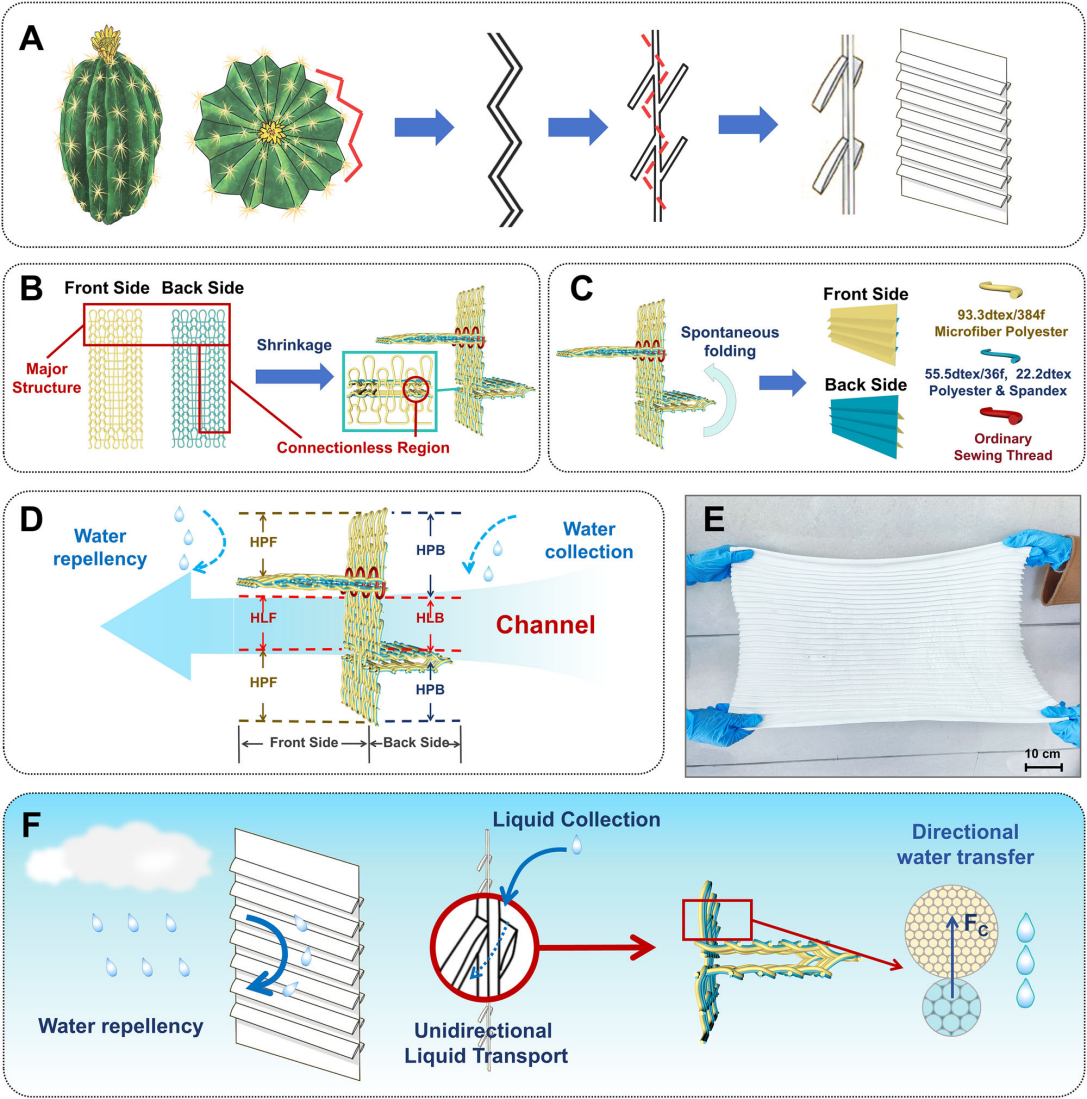
Schematic illustration and fabrication process of an asymmetrical pleated structure textile embedded with a unidirectional water transport channel. (A) Structure design inspired by the pleated surface of cacti. (B) Schematic demonstration of the plated fabric before and after shrinkage. (C) The fabric folded up on its back side while the front side was stitched to create the folding sheet. (D)The finished fabric was divided into hydrophilic (HLF, HLB) and hydrophobic (HPF, HPB) regions, creating a transport channel. (E) Actual image of the asymmetrical pleated structure fabric. (F) The developed fabric demonstrates effective liquid management capacity, including water collection, water transport, and water repellency. The differences in capillary fineness on the two sides achieve a noticeable differential capillary effect.

Herein, inspired by the pleated surface of cacti, a derivative asymmetry pleated structure was designed in this paper by combining the cactus folding structure with differential capillary effect (Figure 1A). Asymmetry pleated structure fabric (APSF), which incorporates a hidden hydrophilic transport channel, has been successfully developed by seamless weft knitting technology. Polydimethylsiloxane (PDMS) and ZnO nanocomposite are selectively sprayed onto the front and back surfaces, while the transport channel remains untreated. The finished APSF is then divided into hydrophobic front/back sides and hidden hydrophilic back/front sides. Notably, the asymmetrical pleated structure of the fabric enhances its water repellent and liquid collecting abilities by utilizing inclined folding sheets and conceal any wetting appearance on the front side (Movie S1). Additionally, the unidirectional water transport property is achieved by configuring yarn materials on this pleated knitted fabric, with microfiber polyester as the top yarn and a wrapped polyester/spandex yarn as the bottom yarn. The distinct linear density of single fibers leads to different capillary fineness, resulting in asymmetric Laplace pressure, so that the liquid can be spontaneously directionally transported [12]. In contrast to natural fibers, water absorption in synthetic fibers relies on the capillary wicking effect instead of forming intermolecular hydrogen bonds in hygroscopic natural fibers after liquid absorption, which is conducive for rapid drying [13]. This innovative textile demonstrates the ability to transport water rapidly, achieving a transfer time of less than 1 s and an impressive AOTI of 499.57%. By integrating water repellency, water collection, and directional water transport into a single cohesive system, the APSF textile presents a significant advancement in moisture regulation technology. Its construction strategy is particularly promising for applications in human comfort, as it efficiently channels sweat away from the skin to the exterior while simultaneously preventing the penetration of external liquids. Overall, this novel design may enlighten us to design high‐performance liquid management materials, with potential applications in fog harvesting [14], flexible microfluidics [15], and oil‐water separation [16].

## 2 Result and Discussion

### 2.1 Wettability, Microstructure, and Chemical Properties of the Fabric

The fabrication procedure of APSF is depicted in Figure 1B,C. On the back side of the fabric, it was evident that the alternately knit coils created a connectionless region, which folded upward automatically upon leaving the knitting machine due to the drawing force of the coils. It can be observed that this folding results in numerous holes distributed along the pleat, derived from the connectionless region. On the front side of the fabric, the folding sheet, with opposite direction, was achieved by stitching. Overall, an asymmetrical folded structure was constructed via coils’ shrinkage, exhibiting a reverse folding orientation on the different sides of the fabric. On the front side, a downward folding sheet is utilized to prevent external liquid intrusion, while on the back side, an upward folding sheet serves the purpose of collecting water. Significantly, the water transport channel would be concealed by the folded part of the fabric to establish a hydrophilic area protected by the outer fabric. Hence, it was crucial for this concealed area to remain uncoated during subsequent processing in order to maintain the fabric's initial hygroscopicity. The APSF, as the initial material, was sprayed with PDMS and ZnO. As demonstrated in Figure S1, the coating solution was prepared. It was necessary to take care to orient the folding gap facing downwards during the spraying on the fabric's back side. This process resulted in the division of the hydrophobic finished fabric into hydrophilic (HLF—the hydrophilic front side; HLB—the hydrophilic back side) and hydrophobic (HPF—the hydrophobic front side; HPB—the hydrophobic back side) regions on each side due to the covering of the folding sheet (Figure 1D). The HLF and HLB constructed a water transport channel, while HPF repelled external water and HPB collected and conducted moisture. The addition of PDMS was able to reduce the surface energy while ZnO constructed a micro‐nanostructure surface, contributing to the achievement of a hydrophobic surface. Figure 1E demonstrates the appearance of the asymmetrical pleated structure fabric, created from basic fabric materials manufactured by a knitting machine. Weft knitting technology is a mature industrial technology with a straightforward processing procedure and the capability for large‐scale production. When the semi‐formed fabric can be directly woven on the machine, followed by the post‐processing steps like sewing and coating, the APSF can be easily mass‐produced. The developed fabric is able to repel water, collect water, and transport water directionally (Figure 1F). The former is achieved by the pleated structure, while the latter is attributed to the fiber materials. The microfiber polyester with a finer individual fiber density is utilized as the top yarn on the external side of the knitting fabric, while the wrapped yarn of polyester and spandex with a higher single fiber density is the bottom yarn arranged on the inner side. The greater the difference between the different linear densities of fiber, the stronger the functionality can be generated. The fabric would contract when leaving the machine due to the existence of spandex, causing a tight structure and consequently increasing the number of capillaries per unit area, which is conducive to strengthen the ability of directional water transport [17]. Therefore, this combination results in distinct differences in capillary fineness on the two sides, achieving a noticeable differential capillary effect. Here, to compare the performances between the planar structure and folded structure, the uncoated hydrophilic region, comprised of a major structure, as a newly independent sample with planar structure, is denoted as MSF (fabric woven with major plain structure) in the following investigation.

**FIGURE 2:**
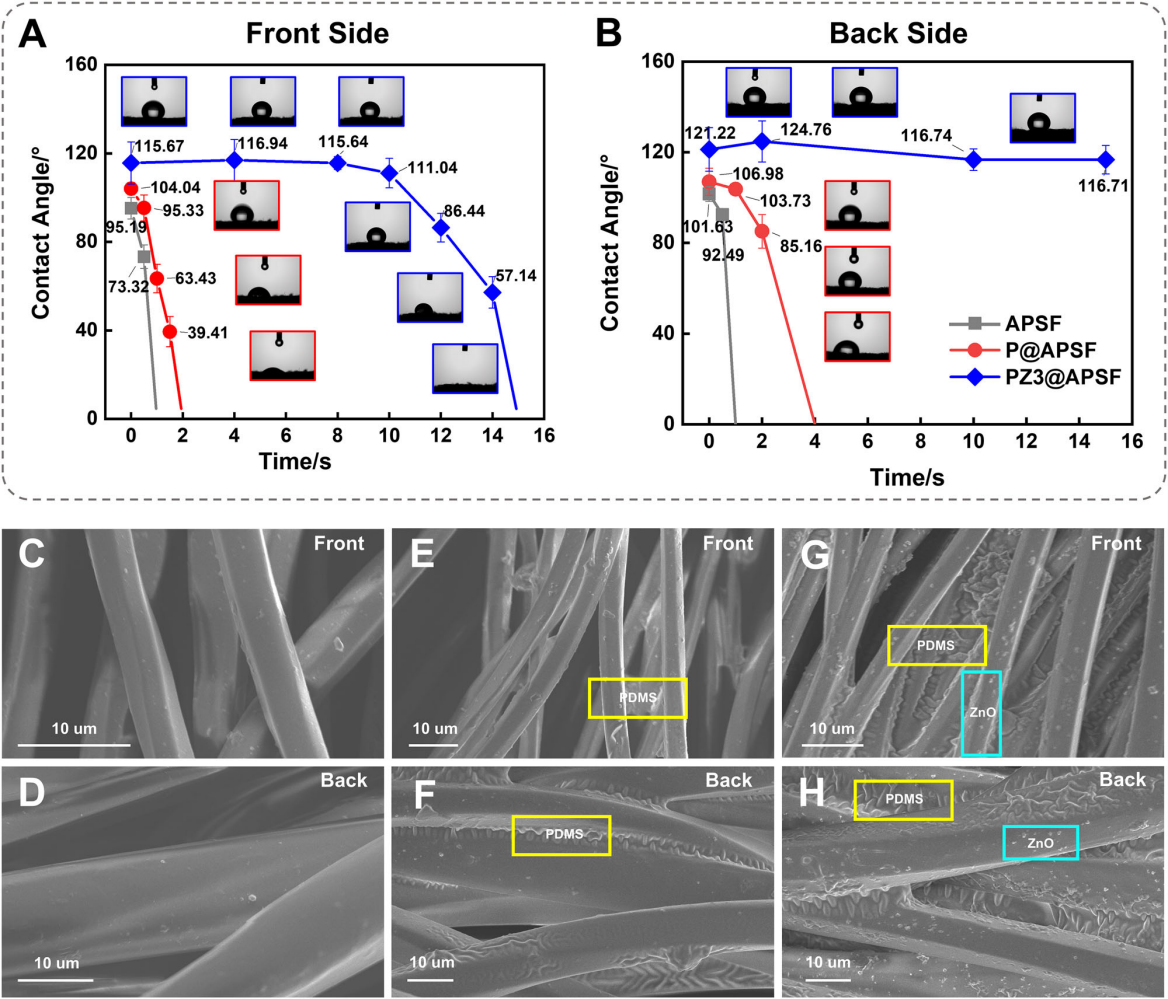
Wetting behavior, microstructure of the fabrics. (A,B) Contact angles of both front (A) and back sides (B) of the fabrics; insets are droplet images when dripped on the fabric. SEM morphologies of pristine fabric (C,D), PDMS finished fabric (E,F), and PDMS/ZnO finished fabric (G,H).

To assess the appropriate gradient humidity, pure PDMS (P@APSF) coated fabric and 0.1%, 0.2%, 0.3%, and 0.4% ZnO and PDMS coated fabrics (PZ1‐4@APSF) were prepared. The wetting behavior of the fabrics before and after the hydrophobic finishing was then tested in Figure 2A,B. The droplet was instantly absorbed within 1 s, whether on the front side or back side, indicating that the pristine APSF showed a contact angle of nearly 0° on both the front and back sides. When water was dripped onto the P@APSF, the water droplet stayed on the surface for 1.5 and 3 s on the front and back sides, respectively, due to the reduction of surface energy as a result of the PDMS coating. Furthermore, PZ@APSF with PDMS and ZnO coating exhibited markedly enhanced hydrophobicity, attributed to the surface nanostructure. As depicted in Figure S2, the contact angles of PZ@APSF on both the front and back sides initially increased and subsequently decreased with the increase of ZnO concentration. For instance, 0.1% ZnO coating brought the contact angle of the back side to 115.13°, which further increased to 117.54° after 0.2%, 121.22° after 0.3%, and then decreased to 117.06° after 0.4%. Moreover, the contact angle on the front side of the fabrics was lower than the back side because of the stronger capillary force generated by microfiber polyester. In PZ3@APSF, the droplet was able to remain steadily for 8 s and more than 15 s on the front and back sides, respectively. The contact angle of PZ3@APSF was also measured after 10 days at room temperature (Figure S3A), showing similar values to the initial day. The droplets of water, Coca‐Cola, and synthetic blood were able to stand steadily on both sides of the surface, indicating the sample's stability properties. Additionally, after 150 min home laundry cycles, the droplet on both sides of PZ3@APSF remained steadily above 15 s on both side (Figure S3B), indicating the stability and launderability of the fabric. Therefore, based on the conclusions of water contact angle, the PZ3@APSF was chosen for further studies.

The SEM morphologies of the fabrics before and after the hydrophobic treatment were examined in Figure 2C–H. Compared with a smooth fiber structure on the pristine fabric, the PDMS coated fabrics exhibited an uneven fiber surface. After adding ZnO nanoparticles to the hydrophobic finishing agent, it was observed that the particles were randomly distributed on the fibers, resulting in a rougher surface. Furthermore, the energy‐dispersive X‐ray spectrometry (EDS) was then used to investigate the distribution of chemical elements. As indicated in Table S1, silicone (Si) content increased to 28% after the hydrophobic treatment of PDMS. And in PZ3@APSF, the addition of ZnO resulted in an increase in zinc (Zn) content to 2%, while the Si content slightly decreased. Figure S4 revealed the presence of Si and Zn elements in the homogeneous dispersion of PZ3@APSF along the fibers, supporting the uniform distribution of hydrophobic finishing throughout the fabric. Furthermore, the coating of PDMS and ZnO had no impact on thermal decomposition in accordance with TGA (Figure S5). Overall, combining the SEM and EDS results, it was confirmed that the PDMS and ZnO were successfully coated on the hydrophobic region of APSF.

### 2.2 Water Management Properties of the Fabric

**FIGURE 3:**
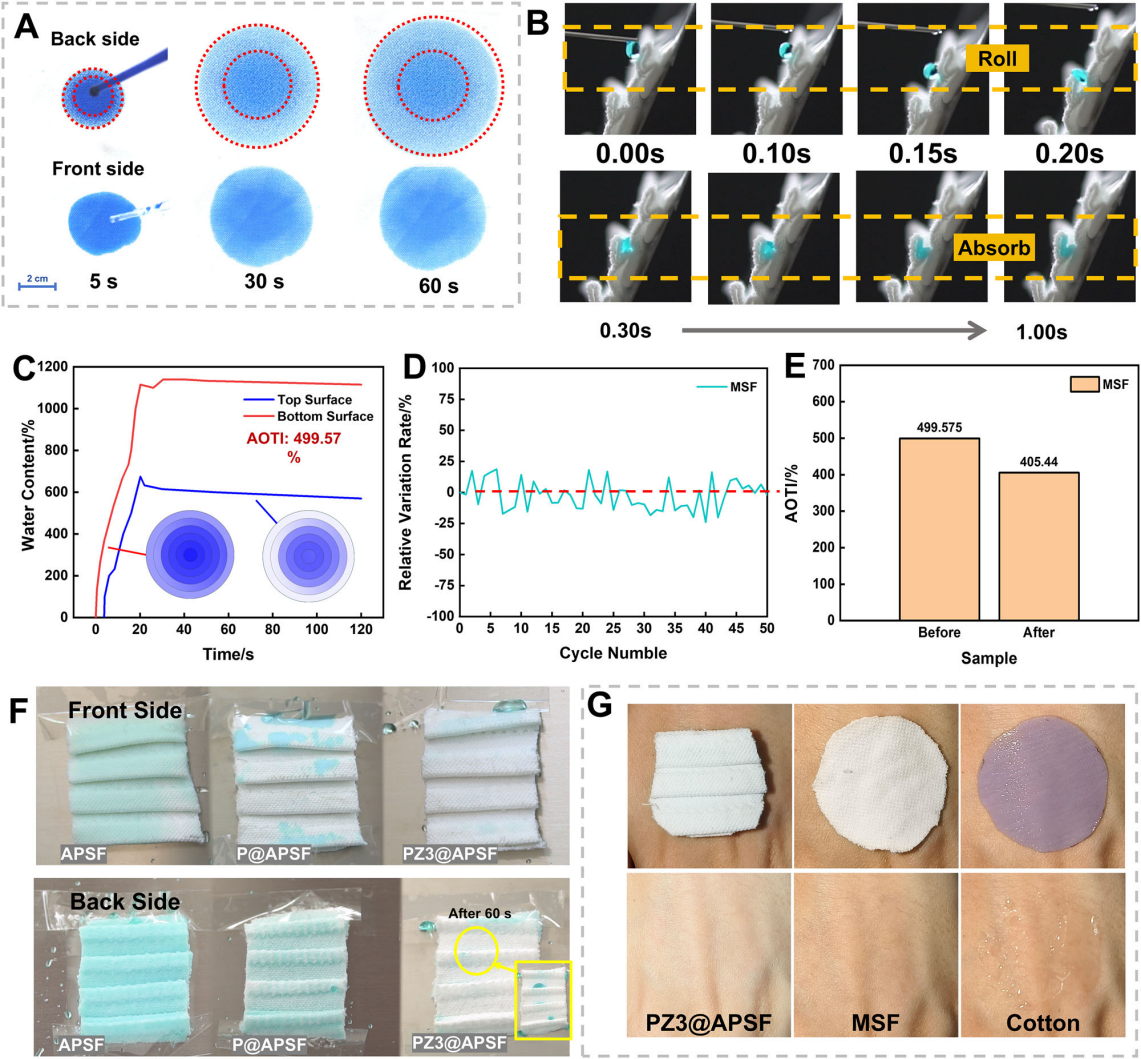
Liquid management properties of the fabrics. (A) Investigation of the unidirectional water transport behavior of MSF by dripping a dyed water drop (0.5 mL) on back side and front side. (B) Still frames taken from videos when water was dripped onto the back side of a hydrophobic finished fabric inclined at 30°. (C) The relative water content on the top and bottom surfaces of the plane structure fabric when water was dropped on the back side. (D) The variation rate of change relative to the initial AOTI value in 50 cycles. (E) The AOTI of MSF before and after the home laundry test for 150 min. (F) The wetting behavior of fabrics after water sprinkling on both the front and back sides. (G) Optical photographs show the quick‐dry performance of the developed PZ3@APSF and MSF compared to the traditional cotton textile.

The water management properties of fabric included directional liquid transport, water repellency, and adhesion. The unidirectional water transport fabric can spontaneously drive the liquid from the inner side (the back side) to the outer side (the front side). As illustrated in Figure 1B, the back side was woven with polyester while the front side was woven with micro polyester, which was able to achieve the fundamental function of the designed fabric. To clarify the capability of directional liquid transport, the water droplets were dropped on the back side of the fabric. As shown in Figure 3A, when the water droplets came into contact with the back side of the MSF, they actively penetrated through it and then spread into the front side, forming layered circles during spreading. When the droplets contacted the front side of the MSF, the water spread only on the front side without showing the pattern of layered circles. The designed fabrics can be locally applied into the garment, especially focusing on high‐sweat volume areas such as the axilla or dorsal region. To simulate the inclination of the garment, in the experiment depicted in Figure 3B, the fabric was positioned at a 30° incline, and a water droplet was dripped on the back side of PZ3@APSF. Upon contact with the HPB region, the droplet did not absorb and then rolled down due to the hydrophobic folded surface, showcasing water capture and transfer capabilities. After that, the droplet was spontaneously absorbed and transported within 1 s after reaching the HLB region—the water transport channel, owing to capillary forces induced by asymmetrical capillary pressure. As regards to P@APSF, similar trends to PZ3@APSF were observed. The water rolled down to the hydrophilic region, followed by absorption. Different from coated fabric, on the APSF without hydrophobic treatment, the liquid was promptly absorbed regardless of the droplet placement on the surface (Movie S2). At the end of the experiment, a comparison of the wetted areas on both sides of these three samples was illustrated in Figure S6. Apart from the hydrophilic region, while PZ3@APSF remained dry, the hydrophobic region of the other fabrics showed partial wetness, suggesting that PZ3@APSF could create a suitable microenvironment. In P@APSF and PZ3@APSF, the channel part that played a role in transporting water was created with the same material of MSF, which was not treated with chemistry, hence, MSF would be used to test the ability of water management, which also represented the ability of P@APSF and PZ3@APSF. To further assess the directional water transport capability of the HL channel in such samples, the MSF with planar structure, consisting of HLF and HLB, was tested by MMT. Figure 3C presents the variations in the relative saline content of the HLF (bottom surface) and HLB (top surface) on MSF during 120 s, respectively. Notably, the water content peaked at 22 s before decreasing, with HLB consistently retaining less water than HLF due to varying capillary effects. The accumulative one‐way transport index (AOTI) of the designed fabric was found to be 499.57%, higher than the other knitting fabric (285.85%) [18], indicating an effective directional water transport performance of textile materials. In comparison to other materials studied in the literature, though the AOTI was lower than those of Janus membranes (higher than 1000%), the hydrophobic finished fabric designed in this work can swiftly transport water within 1 s while others’ transmission time were between 1.5–15 s, demonstrating excellent responsiveness (Table S2). To ensure the long‐term stability of the sample, consecutive tests of the MSF using MMT were carried out. Initially, the AOTI of the sample was measured, followed by drying the sample in cool air using a blow dryer. Subsequently, the variation rate of AOTI after 50 cycles of water droplet tests on the fabric was illustrated in Figure 3D. During the test, the AOTI shows some fluctuation, with between −24% and 20%. With the increase of experiment runs, the transport channel might be blocked by sodium chloride crystals, causing a decrease in numerical value. Overall, the results revealed that the fluctuation of individual relative variation rates can remain by less than 24% for 50 cycles of tests, indicating anticipated long‐term stability. Furthermore, after the home laundry test for 150 min, although the AOTI of MSF was slightly decreased, it remained higher than 400%, signifying the stability and durability of the fabric throughout the reuse process (Figure 3E).

The water repellency of the designed fabric was also experimentally examined via a simplified water‐sprinkling test (Figure 3F). In this test, 5 mL of water held in a curved‐nose spray bottle was randomly sprayed on fabrics placed vertically. The results showed that when water was sprinkled on the front side of PZ3@APSF, it almost completely slid off the fabric. In contrast, APSF and P@APSF were moistened to a certain extent. The water repellent property exhibited on PZ3@APSF probably should be attributed to its low surface energy and micro‐nanostructure surface. Coincidentally, similar trends were observed during the water‐rinsing process on the back side of the fabrics. Strikingly, the tridimensional structure played an essential role in water collection, showing that water was captured in the HLB region of PZ3@APSF. The findings from the water‐sprinkling test confirmed the water repellent property on the front side and the water collection property on the back side of fabric, underscoring that the PDMS&ZnO finished and the asymmetry pleated structure presented a desirable liquid management capability. As regards to the adhesion, the drenched fabrics were placed on the inclined respectively, observing that the cotton fabric was firmly adhered to the plate while MSF and PZ3@APSF rapidly slipped off (Movie S3). Furthermore, the skin adhesion test results on human subjects were demonstrated in Figure 3G to reflect practical usage conditions. It was found that the MSF and PZ3@APSF fabrics outperformed traditional cotton textiles in terms of quick drying due to the presence of asymmetric capillary pores between the two surfaces. This improved drying capability led to a drier and more comfortable feeling on the skin when using the MSF and PZ3@APSF fabrics. This mainly resulted from the polyester with hydrophobicity and the directional water pumping property, leading to lower water content on the back side of the fabric.

The breakthrough pressures of the front and back sides of the designed fabric were also experimentally examined via a water flux test by placing a glass hollow cylinder on either side to hold water (Figure S7A) [19]. The experimental results indicated that PZ3@APSF generated 7 mm H_2_O pressure on the back side and 2.5 mm H_2_O pressure on the front side, whereas APSF and P@APSF failed to generate any significant H_2_O pressure (Movie S4). This outcome is reasonable as the enhanced hydrophobic property of PZ3@APSF would inhibit water to pass through, resulting in a higher breakthrough pressure. In addition, the breakthrough time, defined as the moment when the first drop of water passed through the fabric, was recorded. As illustrated in Figure S7B, the absorption of water in APSF resulted in a longer breakthrough time as the fabric needed to be completely wetted. It is noteworthy that the front side took longer to break through than the back side due to the water‐holding capacity of microfiber polyester. Following the hydrophobic treatment, the breakthrough time of both sides of P@APSF with micro‐hydrophobicity was reduced. Similarly, the breakthrough time for PZ3@APSF was further shortened.

**FIGURE 4:**
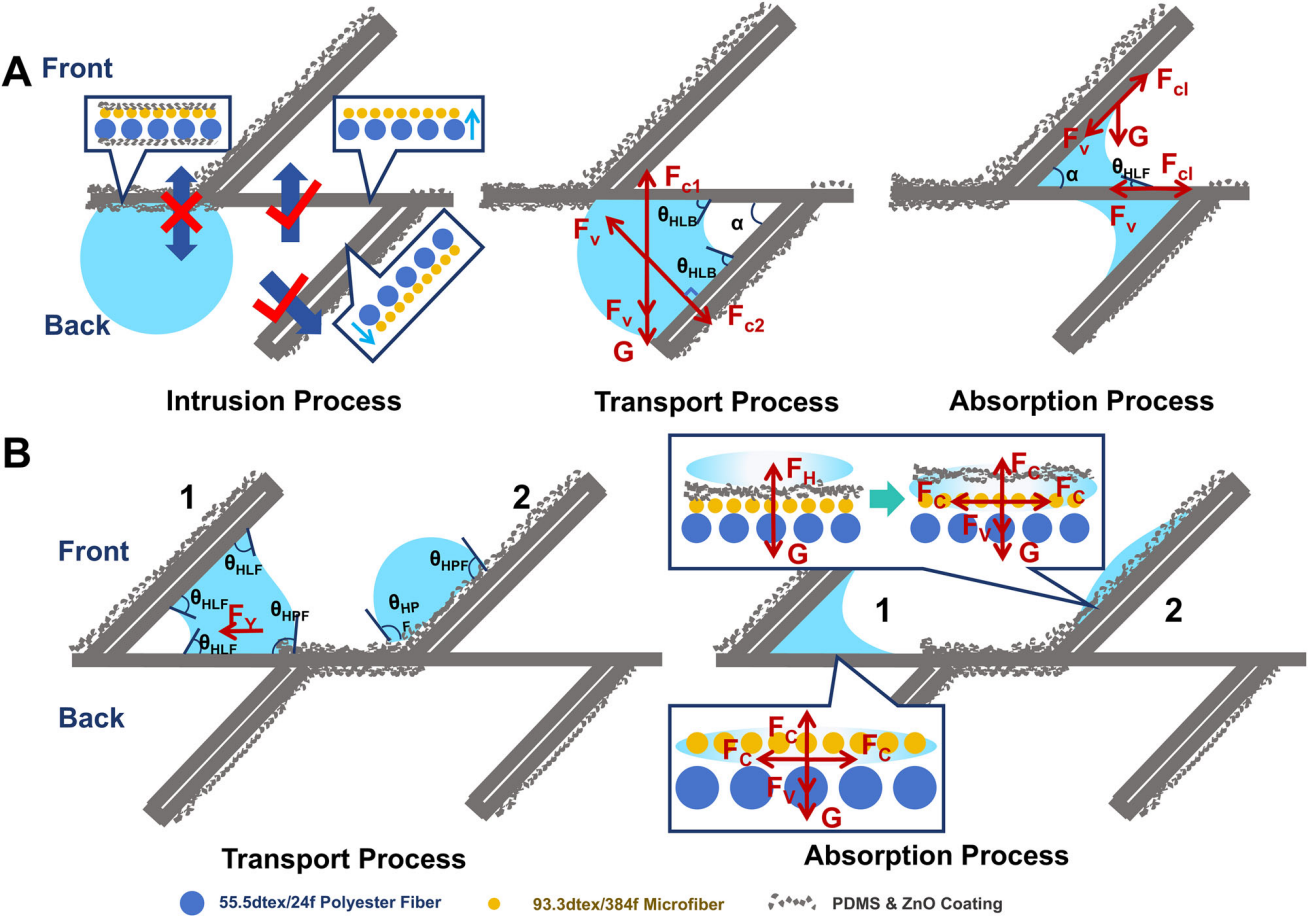
Mechanism of water transport. (A) Illustration of the antigravity water transport through the transport channel when water was dripped on the back side. (B) Illustration of the directional water transport when water was dripped on the front side.

Furthermore, to explain the phenomenon of water management in the designed pleated structure fabric, we investigated the possible mechanism of water droplets passing through the two sides. Figure 4A,B illustrates the behavior when a sweat droplet was dripped on the back side or front side of the horizontally laid fabric, involving three processes: intrusion process, transport process, and absorption process. When a first water droplet is dripped upward to the back side, it can only wet the surface if it comes into contact with the HLB. In the transport process, the water droplet is driven by the capillary force (*F*_c_) arising from the capillary pressure (*P*_c_). Here, the internal structure of yarns was approximately arranged with fibers with circular cross sections arranged in parallel yielding (Figure S8). The HLB, made of 55.5 dtex/36f polyester coils (the radius of fiber = 5.880 × 10^−6 ^m, Text S1), and the HLF, composed of microfiber polyester (the radius of fiber = 2.365 × 10^−6 ^m, Text S1), create a capillary pressure difference (*ΔP*) at the interface. The developed capillary pressure can be estimated in accordance with the Laplace equation:

(1) $$\;P_{c}=\frac{2\gamma \cos\theta }{R}\;$$

(2) $$\Delta P=P_{\mathrm{HLF}}-P_{\mathrm{HLB}}=2\gamma \left(\frac{1}{R_{\mathrm{HLF}}}\cos\theta_{\mathrm{HLF}}-\frac{1}{R_{\mathrm{HLB}}}\cos\theta_{\mathrm{HLB}}\right)$$

where *P*_HLF_ and *P*_HLB_ denote the Laplace pressure of HLF layer and HLB layer, respectively; *R*_HLF_ and *R*_HLB_ are the equivalent radii of the capillary channel on HLF layer and HLB layer, respectively; *γ* is the surface tension of water; the *θ*_HLF_ and *θ*_HLB_ refer to the contact angle of HLF and HLB sides. It is obvious that the capillary pressure in the HLF region is higher than in the HLB region, ensuring sufficient capillary force driven towards the HLF layer (Text S1). As illustrated in Figure 4A, this capillary force (*F*_c1_ = *F*_c2_, due to the same radius of capillary formed by 55.5 dtex/36f polyester) drives the water droplet against gravitational (*G*) and viscous resistance (*F_v_*) forces. In a vertical capillary rise, *F*_c1_ exceeds *F_v_* and *G*, while in an inclined rise, *F*_c2_ overcomes *F_v_*, revealing that the balance of force is independent of folding angle (*α*). Therefore, continuous directional water antigravity transport is achieved in the fabric regardless of the folding angle. During the absorption process, liquid diffusion is limited to the horizontal plane of HLF due to the direction of *ΔP*, preventing vertical liquid transport. Hydrophilic microfibers generate a capillary force (*F*_cl_) driving water diffusion in the HLF region until a balance is achieved with *F_v_* and *G* forces. The equilibrium between these forces is depicted in the following formula:

For the horizontal water absorption process:

(3) $$F_{\mathrm{cl}}-F_{v}=2\pi \gamma \cos\theta_{\mathrm{HLF}}R_{\mathrm{HLF}}-8\pi \mu h\left(t\right)v\;=0$$

For the inclined water absorption process:

(4) $$F_{\mathrm{cl}}\cos\alpha -F_{v}\;\cos\alpha =\left[2\pi \gamma \cos\theta_{\mathrm{HLF}}R_{\mathrm{HLF}}-8\pi \mu h\left(t\right)v\right]\;\cos\alpha =0$$

(5) $$\begin{array}{cc} & F_{\mathrm{cl}}\sin\alpha -F_{v}\sin\alpha -G\;\\ & \;=\left[2\pi \gamma \cos\theta_{\mathrm{HLF}}R_{\mathrm{HLF}}-8\pi \mu h\left(t\right)v\right]\;\sin\alpha -\rho gh\left(t\right)\pi \;R_{\mathrm{HLF}}=0 \end{array}$$

where *α* is the folding angle; *μ* is the water viscosity; *h(t)* is the wicking height; *v* is the wicking velocity. In summary, water will be completely transported to the front side of the fabric, removing excessive liquid.

In the intrusion process of a water droplet on the front side, two conditions emerge: the droplet either rolls down to the HLF surface, denoted as droplet 1, or stands firmly on the HPF surface, referred to as droplet 2 (Figure 4B). In the transport phase, the edge of droplet 1 comes into contact with both the HLF and HPF at the horizontal plane, resulting in a hydrophilic contact angle *θ*_HLF_ and a hydrophobic angle *θ*_HPF_, respectively. Predictably, droplet 1 can move from the hydrophobic to hydrophilic side due to an unbalanced Young's force (*F*_Y_) [20]. As regards droplet 2, it is in a Wenzel state due to the rough surface constructed by ZnO nanoparticles [21], and is subjected to the hydrophobic force (*F*_H_) and gravity (*G*). In the absorption process, the horizontal capillary force emerges immediately, which induces the droplet 1 to spread rapidly in the HLF, while the vertical transport process is obstructed by the capillary pressure difference owing to the smaller radius of the capillary on the HLF layer. Moreover, once *G* is larger than the *F*_H_, droplet 2 initiates penetration into the microfiber capillary, leading to stress distribution analogous to droplet 1. Ultimately, the droplets will be fully absorbed by the hydrophilic microfiber, leaving the back side of the fabric dry.

**FIGURE 5:**
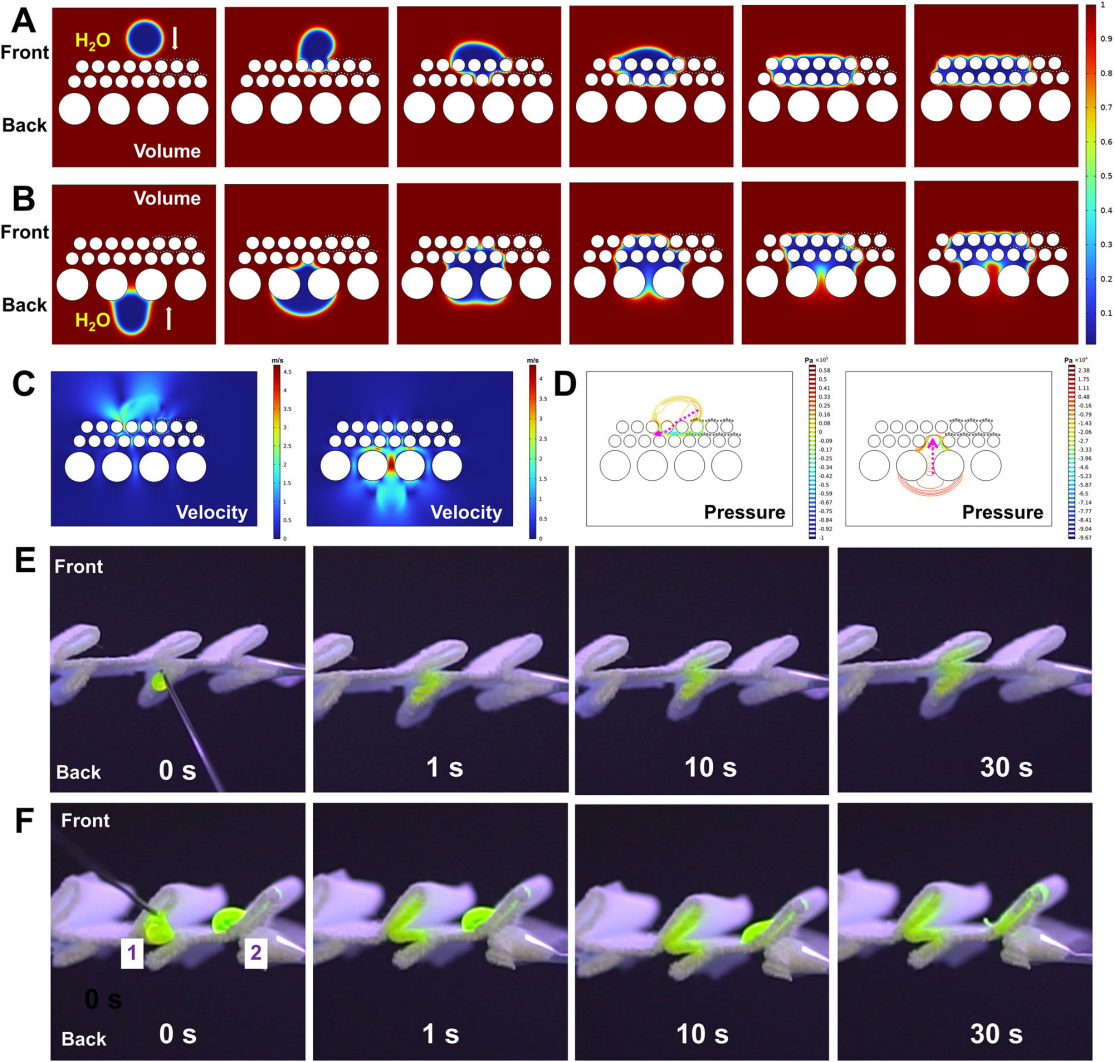
Simulation and verification of water transport. (A,B) Simulation of water movement in the transport channel when water came into contact with the (A) HLF layer and (B) HLB layer of PZ3@APSF using COMSOL software. (C) Velocity distribution of water upon contact with the fabric from the HLF and HLB layers at a specific moment. (D) Pressure distribution of water upon contact with the fabric from the HLF and HLB layers at a specific moment. (E,F) Fluorescence images of the antigravity water transport in the fabric. The water droplet dyed with 0.1 wt% sodium fluorescein contacted the back side and the front side, respectively. The green regions under UV radiation demonstrate the wet areas in the fabric.

To further analyze the directional moisture transport mechanism of the hydrophilic region in PZ3@APSF, COMSOL Multiphysics software was utilized to simulate the fluid field of the water droplet motion process (Text S2). An equivalent simplified cross‐sectional schematic model was established to represent the micro‐structure of microfiber polyester and the fiber of wrapped yarn, depicted as circular cross‐sections of different sizes (Figure S9A). Figure 5A,B illustrates the dynamics of the fluid volume fraction over time as the water droplet was in contact with the HLF and HLB layers of PZ3@APSF. Notably, the simulation results closely aligned with the mechanism of water droplets passing through the two sides. The hydrophobic coating created an imbalance in force, propelling water toward the HLF region, while capillary force prevented backflow when water dripped on the HLF layer. Additionally, Figure 5C,D display the velocity and pressure distribution of water upon contact with the fabric from the HLF and HLB layers at a specific moment. It was observed that water traveled fast from the HLB layer to the HLF layer upon contact with HLB. Moreover, water tended to move away from the hydrophobic coating area, preferentially being absorbed by the HLF layer. When the droplet was dripped on the HPF/HPB of the pleated structure fabric, the droplet was unable to penetrate the hydrophobic coating on the pleated structure fabric, thereby blocking water absorption in the HPF/HPB region (Figure S9B,C). Consequently, the moisture transport capability of PZ3@APSF in a unidirectional manner was further validated through simulations conducted using COMSOL Multiphysics software.

A simplified demonstration model was constructed to validate the transport mechanism discussed above. In this model, a water droplet dyed with 0.1 wt% sodium fluorescein was used to track its transmission trajectory (Figure 5E,F and Movie S5). As shown in Figure 5E, PZ3@APSF was laid horizontally. When the green water droplet was fed upward to contact with the HLB of the fabric, it was driven by the capillary force and transported against gravity. During the initial 1 s, water was observed to flow along the inclined folded sheet due to the lower resistance in the inclined direction compared to the vertical direction. Subsequently, vertical transport of water became noticeable as time progressed, confirming the effectiveness of directional water management in the hydrophilic region. Figure 5F shows two potential conditions for the top side of PZ3@APSF. In one scenario, droplet 1, upon contact with the HLF area, was rapidly absorbed by the microfiber within 1 s. However, it was hard for the liquid to diffuse to HLB because of the repulsive capillary force. In another scenario, the droplet 2 remained on the HPF for more than 10 s. Only after breaking through the hydrophobic layer formed by the nanoparticles could the water be absorbed. Although the liquid permeated the fabric, it was unable to transfer to the back side, ensuring dryness on the inner surface.

### 2.3 Thermal Management, Antimicrobial Property, and Permeability of Fabrics

**FIGURE 6:**
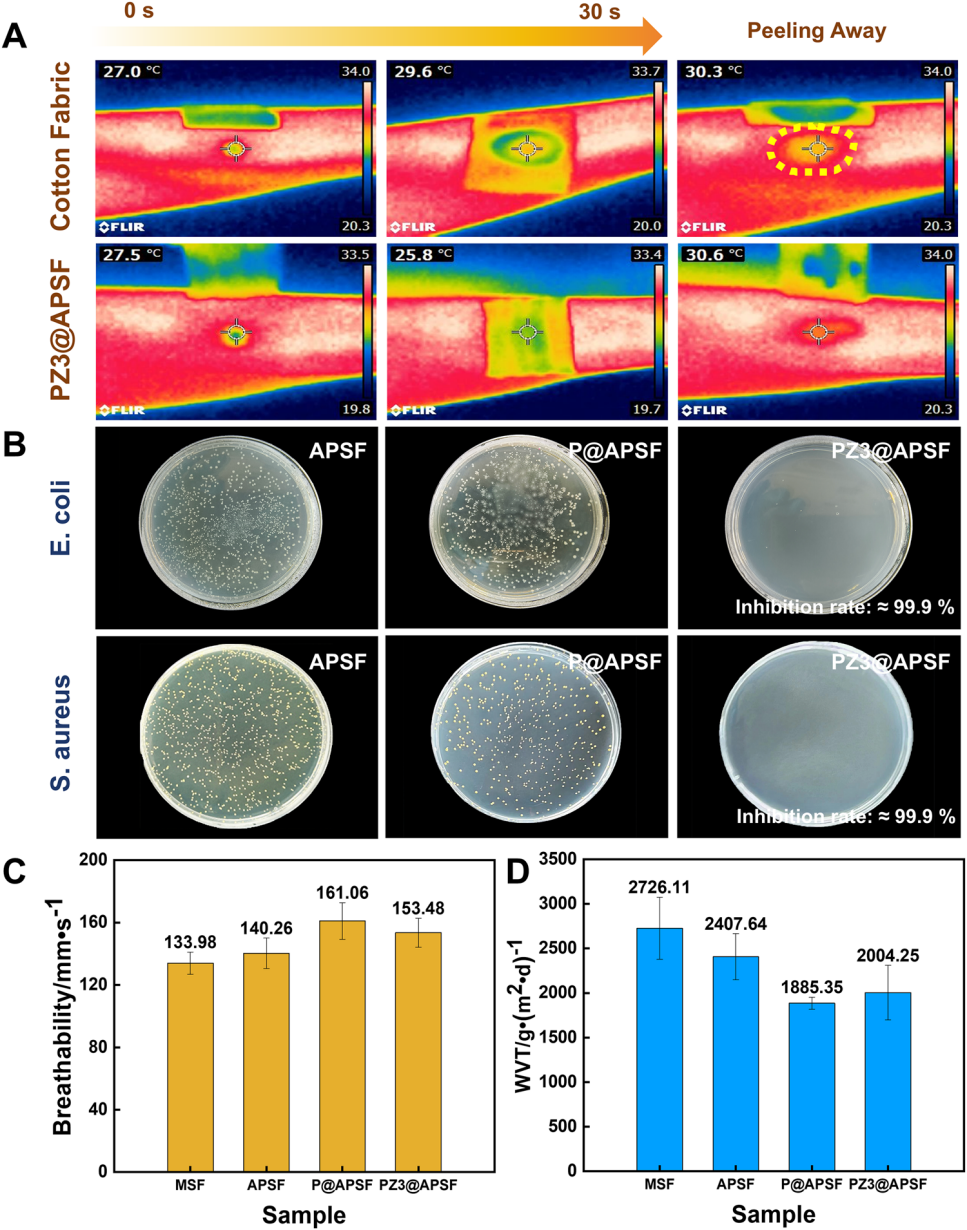
Thermal management, antimicrobial property, and permeability of fabrics. (A) Infrared camera images of the textiles placed on the skin. (B) Antimicrobial property of fabrics before and after hydrophobic treatment. (C) Air permeability of fabrics before and after hydrophobic treatment. (D) Water vapor transmission rate of fabrics before and after hydrophobic treatment.

In addition to liquid management, thermal management, antimicrobial property, and permeability were also considered. Thermal management property was evaluated by recording the surface temperature using infrared thermal images. The samples were first placed on the skin surface, and then the water was dropped between the skin and fabric to simulate perspiration. Cotton fabric, which dissipates heat passively while sweating, was used to compare the influence of sweat absorption and water evaporation. As depicted in Figure 6A, after a 30 s water diffusion period, skin temperature under PZ3@APSF was higher than that under conventional cotton fabric, and the cold sensation area was smaller. These findings indicate the efficient regulation of the skin surface microenvironment through directional water transport abilities. Additionally, the antimicrobial properties of PZ3@APSF, attributed to ZnO nanoparticle coating, were confirmed by a bacterial killing ratio of 99.9% for *Staphylococcus aureus* and *Escherichia coli* (Figure 6B). In contrast, P@APSF hardly possessed antibacterial activity. This phenomenon is explained by the reactive free radicals generated by metal oxide ZnO, which deactivate intracellular proteases in bacteria when exposed to external light [22]. The capability of bacteria to kill the fabric makes it possible to be applied in medical textiles. Figure 6C revealed that the asymmetrical pleated structure and hydrophobic finishing did not have a serious detrimental effect on the breathability. Although the asymmetrical pleated structure fabric is thicker than traditional flat structure textiles, the air can penetrate through the fabric due to the creation of pores in the connectionless region. While the water vapor transmission rate (WVT) exhibited a contrasting trend, with the obstruction of the pleated structure resulting in a lower value for APSF compared to MSF (Figure 6D). Furthermore, the application of the hydrophobic finishing agent led to a decrease in moisture permeability. Despite the reduction in WVT, fabric‐matrix demonstrated superior performance over membrane‐matrix (1400 g m^−2 ^d^−1^) [23], displaying potential for functional materials. Based on the above‐mentioned factors, the asymmetrical pleated structure fabric coated with PDMS & ZnO was undoubtedly to be a relatively promising functional material.

### 2.4 The Potential Applications of APSF

The developed fabrics have shown excellent comprehensive properties, especially the short transmission time (<1 s) and high AOTI (499.57%). In contrast to the previous studies, with approximately 15 s of transmission time, APSF can swiftly remove the sweat as soon as possible while perspiring, ensuring the dryness of skin and maintaining a comfortable micro‐environment for humans. Additionally, APSF is able to achieve large‐scale production through seamless knitting technology, demonstrating good feasibility.

However, there are still some issues that should be overcome, such as the thickness of this fabric. The pleated fabric is thicker than those with a plat structure due to its multi‐layer structure, causing a challenge on heat convection, although it can also dissipate heat through evaporation. Therefore, there will be some progress in our future research, improving the structure with microcosmic design to reduce thickness while retaining performance with water repellency, water collection, and directional water transport.

**FIGURE 7:**
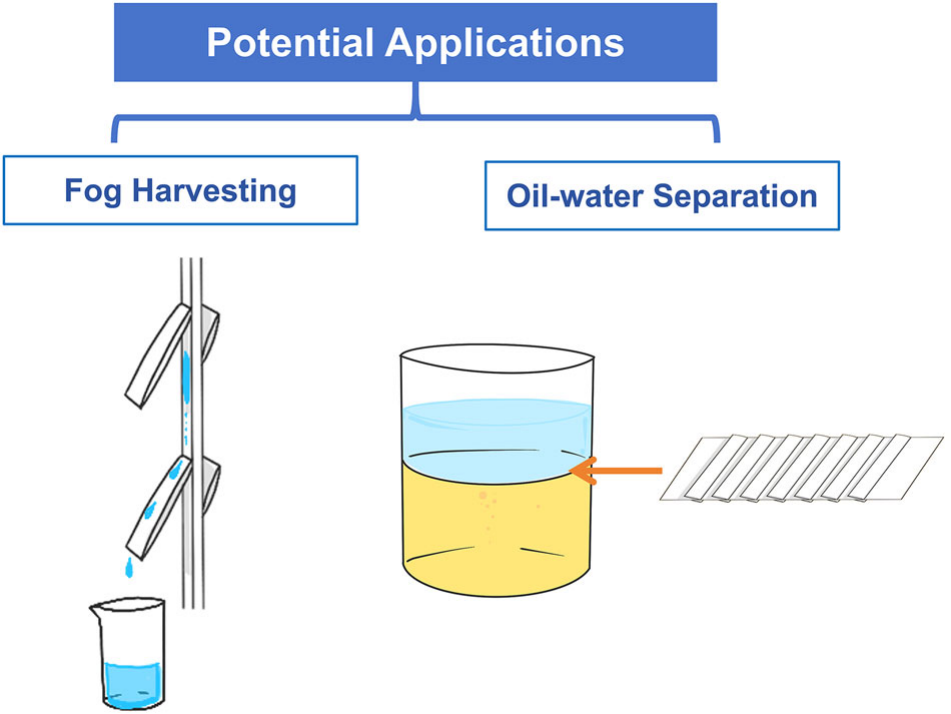
The potential applications of APSF.

Overall, such designed fabric has great potential for application which can be utilized not only in functional clothing but also in fog harvesting and oil‐water separation (Figure 7). For instance, in fog harvesting, the upward folded sheet of the fabric efficiently collects moisture which is then transported to the outside. Therefore, this functional fabric shows significant promise for integration into smart textiles, expanding its applications to various industrial applications.

## 3 Conclusion

In summary, we demonstrate that an asymmetrical pleated structure textile embedded with a unidirectional water transport channel, featuring a hydrophobic modified back/front surface, can achieve synergistic liquid management. The managing processes include water collection, directional water transport (with an accumulative one‐way transport index of 499.57 %), and water repellency (with a stable contact angle exceeding 120°). The liquid autonomously moves through the transport channel due to the capillary force, while the inclined surfaces are hydrophobic, treated using PDMS and ZnO. After the home laundry test for 150 min, the AOTI of MSF remained higher than 400%, proving the stability and durability of the fabric throughout the reuse process. The transport channel can efficiently transport sweat from the skin side to the exterior while the outside surface is able to prevent the permeation of external liquids, ensuring individual safety and comfort. This design has the potential to broaden the applications of such materials, providing innovative approaches in the field of fog harvesting, flexible microfluidics, oil‐water separation, and energy conservation.

## 4 Experimental Section

### 4.1 Materials

The wrapped yarn of polyester and spandex (55.5 dtex/36f, 22.2 dtex) was obtained from Zhuji Huashu Chemical Fiber Co., Ltd., China. The microfiber polyester (93.3 dtex/384f) were supplied by Qingdao Lianrunxiang Textile Technology Co., Ltd., China. The PDMS (Sylgard 184) and curing agent were obtained from Dow Corning (MI). The ZnO nano‐particles (200 nm) was purchased from Hebei Yuanying New Material Co., Ltd., China. The n‐hexane (AR) was supplied by Guangdong Baisi Chemical Technology Co., Ltd., China. Synthetic blood (surface tension = 0.042 ± 0.002 N m^−1^, pH value = 7.3 ± 0.1) was purchased from Fuzhou Phygene Bio‐Technology Co., Ltd., China. *Escherichia coli* and *Staphylococcus aureus* were provided by Shanghai Luwei Technology Co., Ltd., China.

### 4.2 Fabrication of APSF

The plated knitting fabric was produced on a seamless underwear circular knitting machine (SM8‐TOP2 MP2, Santoni (Shanghai) Knitting Machinery Co., Ltd., China) with a gauge of 28, a cylinder diameter of 38.1 cm, and a total stitch count of 1344. The microfiber polyester was set as the top yarn while the wrapped yarn of polyester and spandex was set as the bottom yarn. The folded structure of the front side was produced employing a sewing machine (A3, Jack Technology Co., Ltd., China).

### 4.3 Fabrication of PZ@APSF

To begin with, 3.0 g of PDMS and 0.3 g of curing agent were dissolved in 100 mL of n‐hexane with ultrasonic treatment for 1 h at room temperature to form a homogeneous dispersion. Subsequently, ZnO at varying concentrations of 0.1%, 0.2%, 0.3%, and 0.4% was added to the resultant solution, and ultrasonic dispersion at room temperature was carried out until complete dissolution of ZnO. Then, the coating solution, prepared as mentioned, was evenly sprayed on both sides of the fabric using an electric spray pen, with a coating density of 0.16 mL cm^−2^ and spraying speed of 10 mL min^−1^. Importantly, to preserve the initial hydrophilic channel, the folded structure was flattened using a steam ironing machine to avoid the coating of the interior hydrophilic region. The spraying process was conducted at a temperature and relative humidity of 20 ± 2°C and 60 ± 2%, respectively. After that, the coated fabrics were dried at 50°C for 5 h in an oven. Finally, the asymmetrical pleated fabrics structure coated with PDMS and ZnO were obtained, abbreviated as PZ1@APSF, PZ2@APSF, PZ3@APSF, and PZ4@APSF, respectively.

### 4.4 Fabrication of P@APSF

First, a homogeneous dispersion was formed by dissolving 3.0 g of PDMS and 0.3 g of curing agent in 100 mL of *n*‐hexane at room temperature, followed by ultrasonic treatment for 1 h. Subsequently, the solution was utilized for spray treatment. The detail of the coating was the same as PZ@APSF. Finally, the resulting fabric with an asymmetrical pleated structure, coated with PDMS, was denoted as P@APSF.

### 4.5 Characterizations

The morphological structure of the APSF, P@APSF and PZ@APSF was characterized by the scanning electron microscope (SEM) (SU 1510, Hitachi High‐Technologies Corp., Tokyo, Japan) combined with energy‐dispersive X‐ray spectrometry (EDS). A thermogravimetric analyzer (TGA‐Q500, TA Instruments, Waters, USA) was used to investigate the thermal decomposition at a heating rate of 20°C min^−1^ from 25 to 700°C with nitrogen gas medium.

A YG461E‐III automatic gas permeation instrument (Ningbo Textile Instrument Factory, China) was used to investigate the breathability of textiles according to the standard GB/T 5453−1997. WVT was detected by the moisture permeability testing apparatus (YG601H‐II, Ningbo Textile Instrument Factory, China) in accordance with GB/T 12704.2−2009 standard. The front side of fabric contacted with the atmosphere while the back side was toward the water in the assembled dish. The measurement was conducted at a temperature, relative humidity, and a wind speed of 38 ± 2°C, 50 ± 2%, and 0.3 – 0.5 m s^−1^, respectively. The water contact angle of the samples was characterized using an optical contact angle meter system (JC2000DM, Beijing Zhongyi Kexin Technology Co., Ltd. China). The home laundry test was carried out using a laundry machine with ten stainless steel balls and 0.4% WOB reference detergent (Shanghai Mien Testing Instrument, Co., Ltd., China). The test lasted approximately 150 min, after which the washed fabrics were air‐dried at room temperature. An infrared thermal camera (FLIR‐E6390, Estonia) was used to capture the surface temperature of the human body, including images of the fabric in its initial dry state, images of wetting for 30 s, and images of the skin after removing the fabric.

### 4.6 Investigation of the Water Management

The visual droplet dripping test was conducted on a homemade right triangle frame. Water droplets were dripped onto the back sides of the 30° inclined laid fabric (5 × 8 cm) after hydrophobic treatment (Figure S10). The images were recorded by the digital camera (SONY, ILCE‐A6300). In addition, a moisture management tester (MMT, Q290, Xian Weierze Instrument Technology Co., Ltd., China) was employed to further assess the capability of unidirectional water transport based on GB/T 21655.2‐2009 standard. The experiment was carried out by recording the changes in water content on both sides of the fabric sample over a 120 s period and subsequently generating the curve of the relationship between the water content difference and time, referred to as the accumulative one‐way transport index (AOTI). To observe the water repellent performance of the fabric, it was vertically mounted on a board. A curved‐nose spray bottle holding 5 mL of water was used to wet the fabric. The fabric's wetting condition was then recorded both before and after spraying. Moreover, the antigravity directional water transport process was performed under UV radiation (*λ* = 365 nm). The fabric was placed horizontally on the frame. A volume of 10 µL water droplet (0.1 wt% sodium fluorescein) was supplied upward to the back surface and downward to the front surface of the fabric. The digital camera (SONY, ILCE‐A6300) was used to track the transport of water. The adhesion of fabric in the wet state was evaluated by the slide condition on the triangle frame. The samples were first soaked in 10 mL of water for 1 min and then allowed to naturally dry until no water was dripping. Subsequently, the fabric was placed on the inclined plane immediately to observe whether it could slide down. Notably, the back side of the fabric was set to contact the plate. Additionally, a homemade device was set up to measure the breakthrough pressure of the fabrics. The device comprised a glass tube with the bottom end contact with the testing fabric, a water source from a syringe pump, and a glass bottle collector underneath. The fluid rate during the test was set to 1 mL min^−1^. The breakthrough pressure was recorded as the pressure at which the water initiates passing through the fabric.

### 4.7 Measurement of the Antimicrobial Properties

The antimicrobial activity assessment was conducted in accordance with the GB/T 20944.3‐2008 standard, utilizing *E. coli* and *S. aureus a*s the experimental bacteria. Initially, bacterial suspensions were prepared by inoculating 3–10 generations of bacteria into 20 mL of nutrient broth and incubating them for 18–20 h at 37°C and 130 r min^−1^. Subsequently, the concentration of the bacterial solution was quantified and adjusted to range from 0.7 × 10^9^ to 0.9 × 10^9^ CFU mL^−1^, as determined through spectrophotometry. Thereafter, the bacterial suspension was sequentially diluted by ten times using the nutrient broth and further diluted by 100 times using PBS. The concentration of the bacterial suspension in direct contact with the sample ranged from 0.7 × 10^6^ to 0.9 × 10^6^ CFU mL^−1^. Then, the bacterial suspension was mixed with the fabric samples in a conical flask and cultured in a constant‐temperature oscillator at 24°C and 150 r min^−1^ for 18 h to ensure complete contact between the sample and the bacterial suspension. Finally, a 1000‐fold‐diluted bacterial suspension was pipetted into a solid Petri dish and coated uniformly. The Petri dish was then placed in a 37°C constant‐temperature incubator for 24 h.

## Conflicts of Interest

The authors declare no conflict of interest.

## Supporting information




**Supporting File 1**: exp270066‐sup‐0001‐SuppMat.docx

**Supporting Movie 1**: exp270066‐sup‐0002‐MovieS1.mp4

**Supporting Movie 2**: exp270066‐sup‐0003‐MovieS2.mp4

**Supporting Movie 3**: exp270066‐sup‐0004‐MovieS3.mp4

**Supporting Movie 4**: exp270066‐sup‐0005‐MovieS4.mp4

**Supporting Movie 5**: exp270066‐sup‐0006‐MovieS5.mp4

## Data Availability

The data that support the findings of this study are available from the corresponding author upon reasonable request.
