# Supplementary material for: Bioinspired Asymmetrical Pleated Textile With Unidirectional Transport Channel for Personal Moisture and Thermal Management
Source: Exploration (Beijing). 2025 Jun 12;5(5):20240357. doi: 10.1002/EXP.20240357 (PMC12561288; doi:10.1002/EXP.20240357)
Supplement: Supplementary file 1 — Supporting File 1: exp270066‐sup‐0001‐SuppMat.docx [file EXP2-5-20240357-s002.docx]

**Supporting Information for Review Only**

**Bioinspired Asymmetrical Pleated Textile** **with Unidirectional Transport Channel for Personal Moisture and Thermal Management**

Meitong Ge^a^, Fengxiang Chen^b^*, Chaoyu Chen^a^, Honglian Cong^a^, Xin Wang^c^, Zhijia Dong^a^*, Pibo Ma^a^*

a. Engineering Research Center of Knitting Technology, Ministry of Education, College of Textile Science and Engineering, Jiangnan University, Wuxi 214122, China

b. State Key Laboratory of New Textile Materials and Advanced Processing Technologies/National Local Joint Laboratory for Advanced Textile Processing and Clean Production/Hubei Key Laboratory of Digital Textile Equipment, Wuhan Textile University, Wuhan 430200, P.R. China

c. School of Fashion and Textiles, RMIT University, Melbourne 3056, Australia

*Corresponding author:

fxchen_czx@wtu.edu.cn; [dongzj0921@jiangnan.edu.cn](mailto:dongzj0921@jiangnan.edu.cn); [mapibo@jiangnan.edu.cn](mailto:mapibo@jiangnan.edu.cn)

**Movie Captions.**

**Movie S1.** The presentation of liquid transport on the asymmetrical pleated structure fabric

**Movie S2.** The water transport experiment tested by dripping droplets onto the back side of fabrics

**Movie S3.** The adhesion of liquid-saturated fabrics including cotton fabric, MSF and APSF

**Movie S4.** Continuous water flux (1 mL/min) by a needle to test the breakthrough pressure on the front and back sides of the PDMS/ZnO finished fabric

**Movie S5.** The experiment of water transport under UV

**Fig.S1**

**
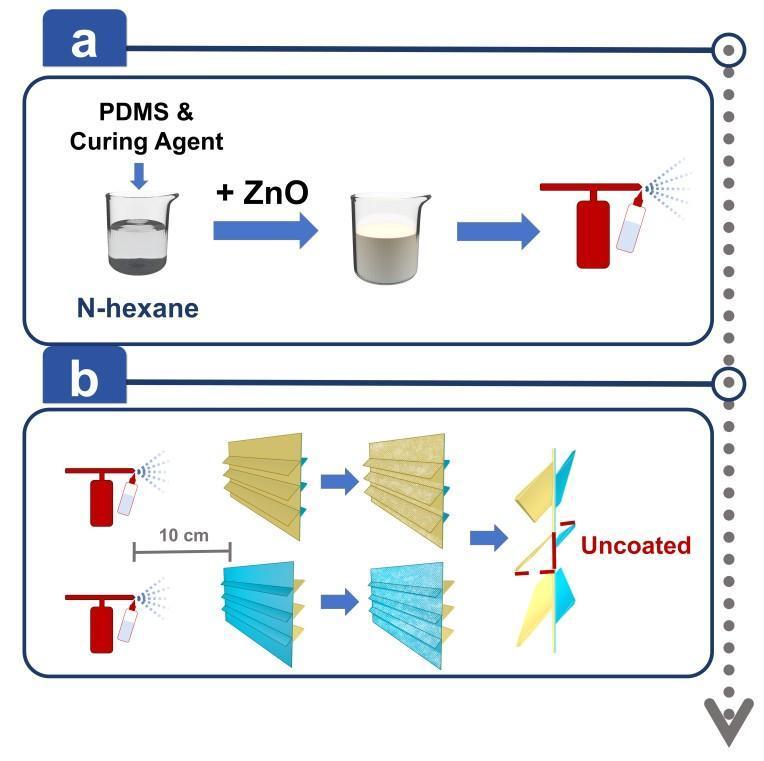
**

**Fig.S1.** a) The preparation of PDMS/ZnO resultant solution. b) The fabric was sprayed on the both sides using hydrophobic finishing agent.

**Fig.S2**


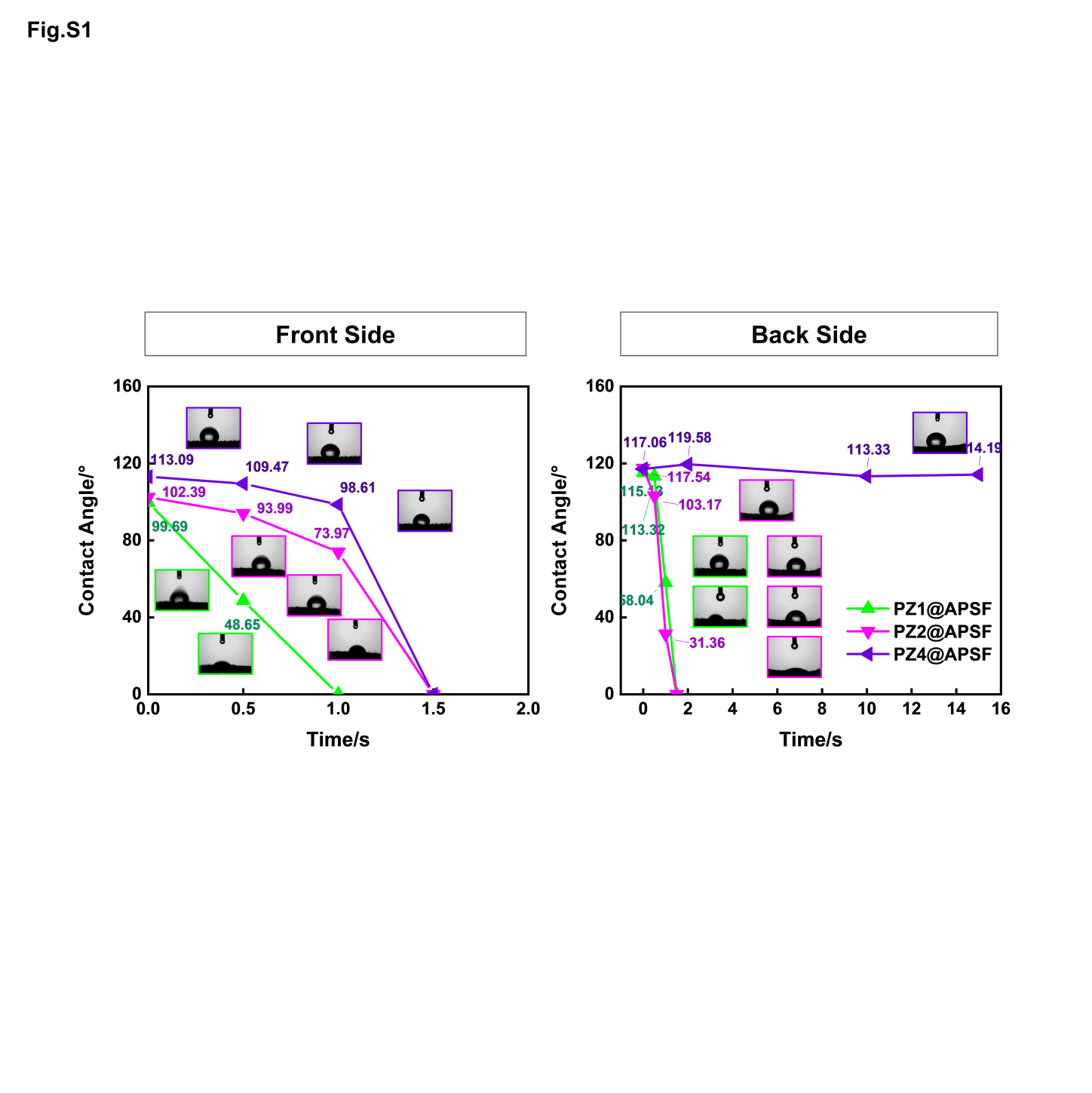


**Fig.S2.** Contact angles of both the front and back sides of the hydrophobic finished fabric change as the ZnO concentration increases.

**Fig.S3**

**
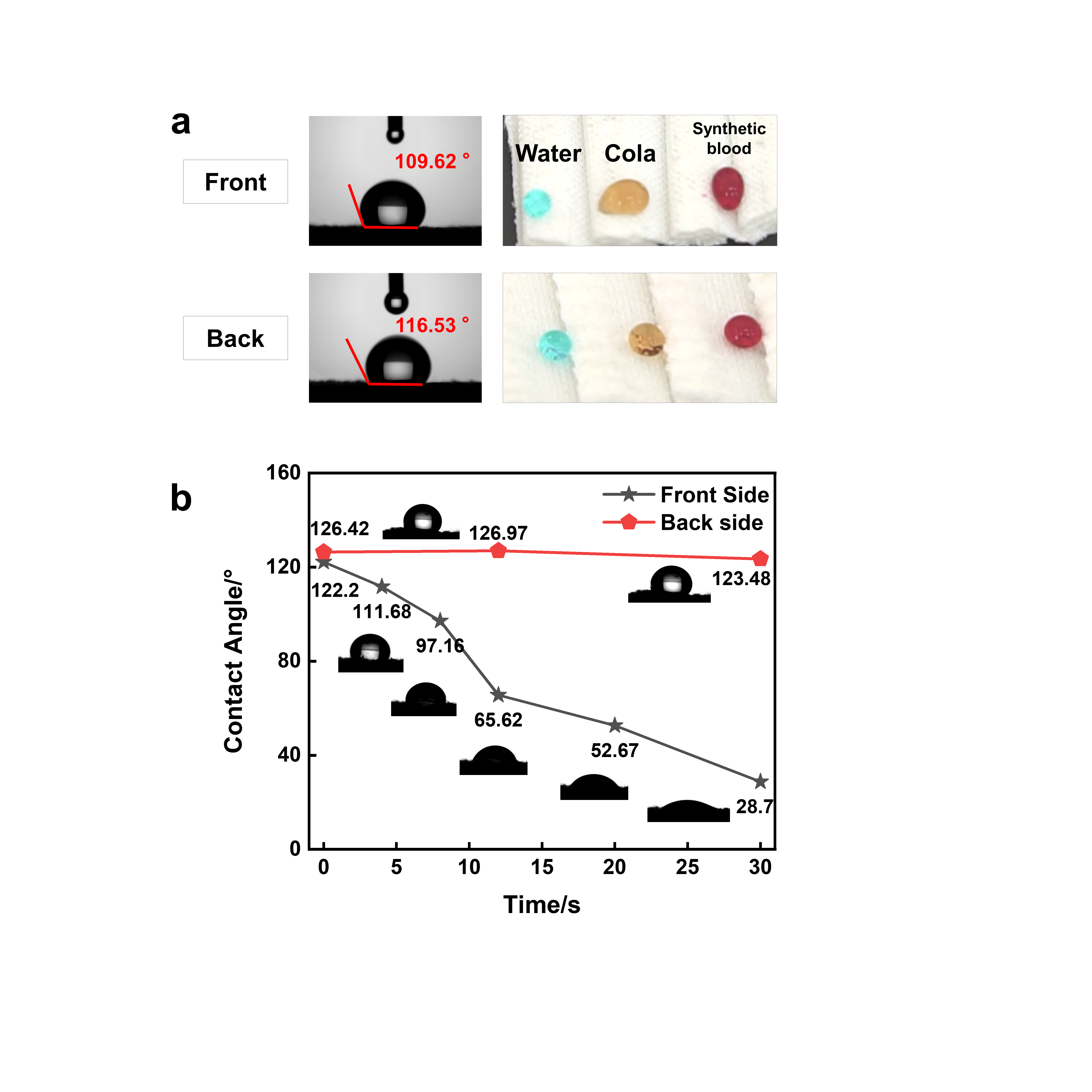
**

**Fig.S3.** a) Contact angles of both the front and back sides of the hydrophobic finished fabric after PDMS/ZnO coating for 10 days and droplet images when dripped on the both sides of fabric. b) Contact angles of PZ3@APSF after 150 min home laundry test.

**Fig.S4**


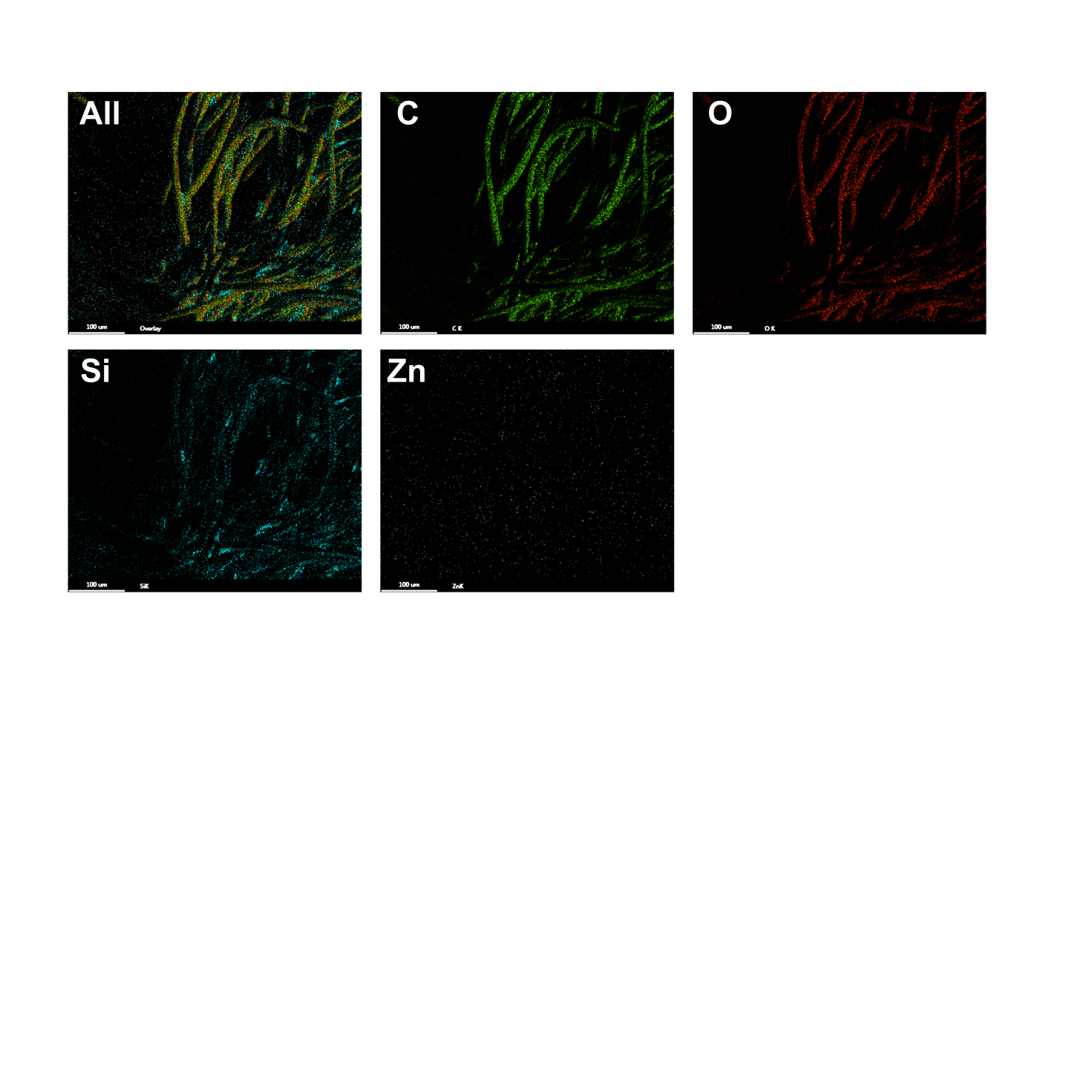


**Fig.S4.** Distribution of elements in a hydrophobic fabric treated with PDMS/ZnO.

**Fig.S5**


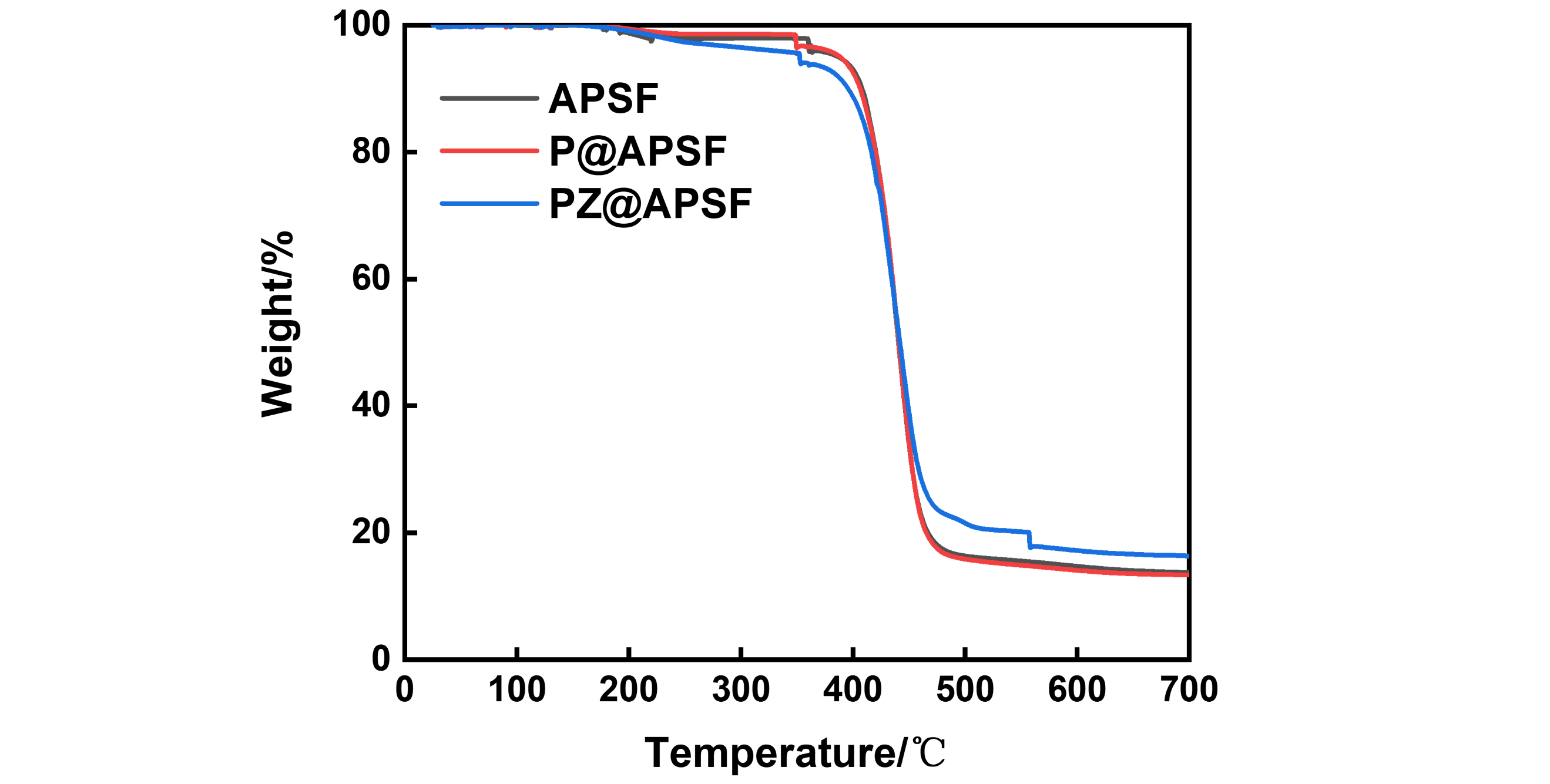


**Fig.S5.** Thermogravimetric analysis (TGA) spectra of pristine polyester fabric, hydrophobic fabric treated with PDMS, and hydrophobic fabric treated with PDMS/ZnO.

**Fig.S6**


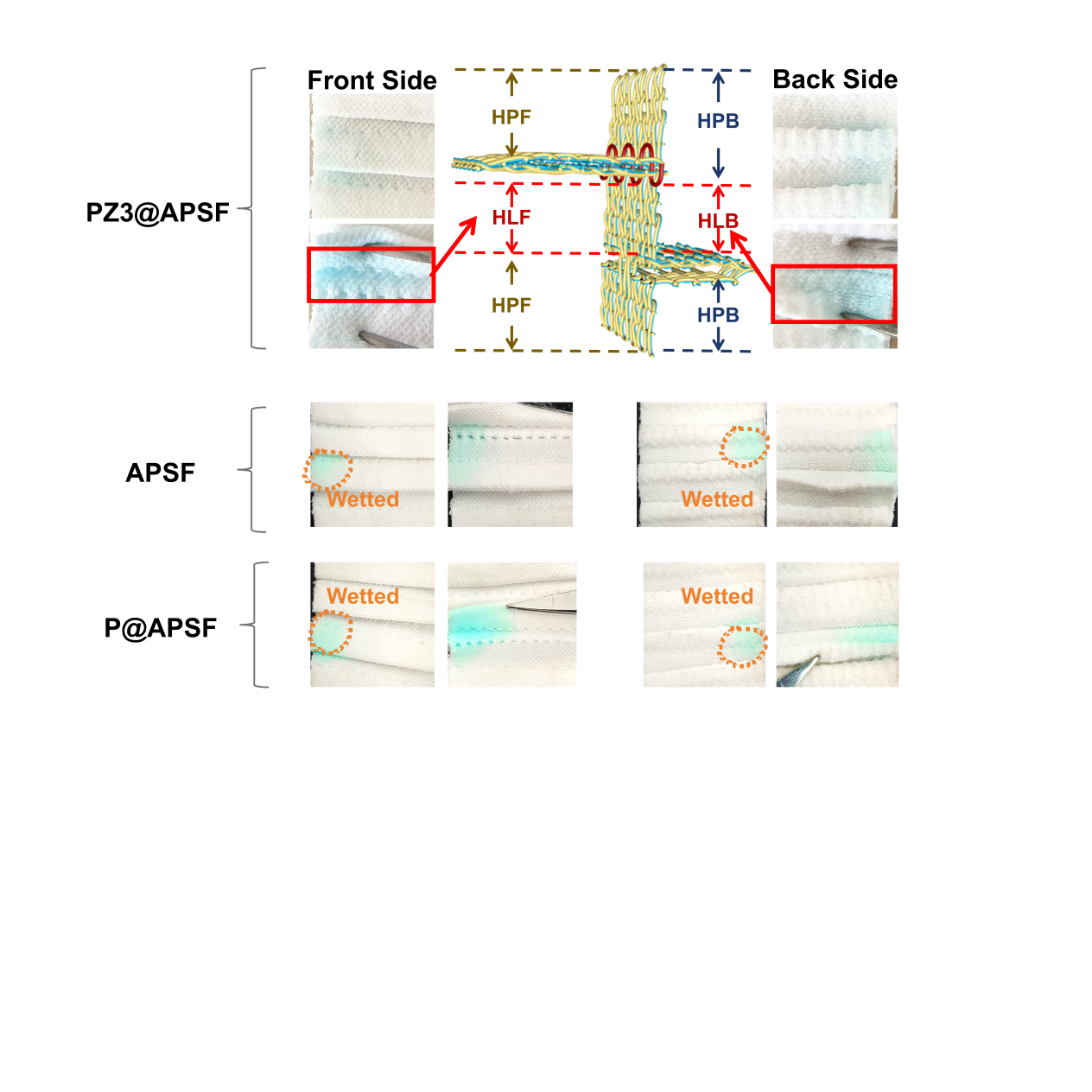


**Fig.S6.** Wetted areas after water dripping experiment on the back side of pristine polyester fabric, hydrophobic fabric treated with PDMS, and hydrophobic fabric treated with PDMS/ZnO.

**Fig.S7**


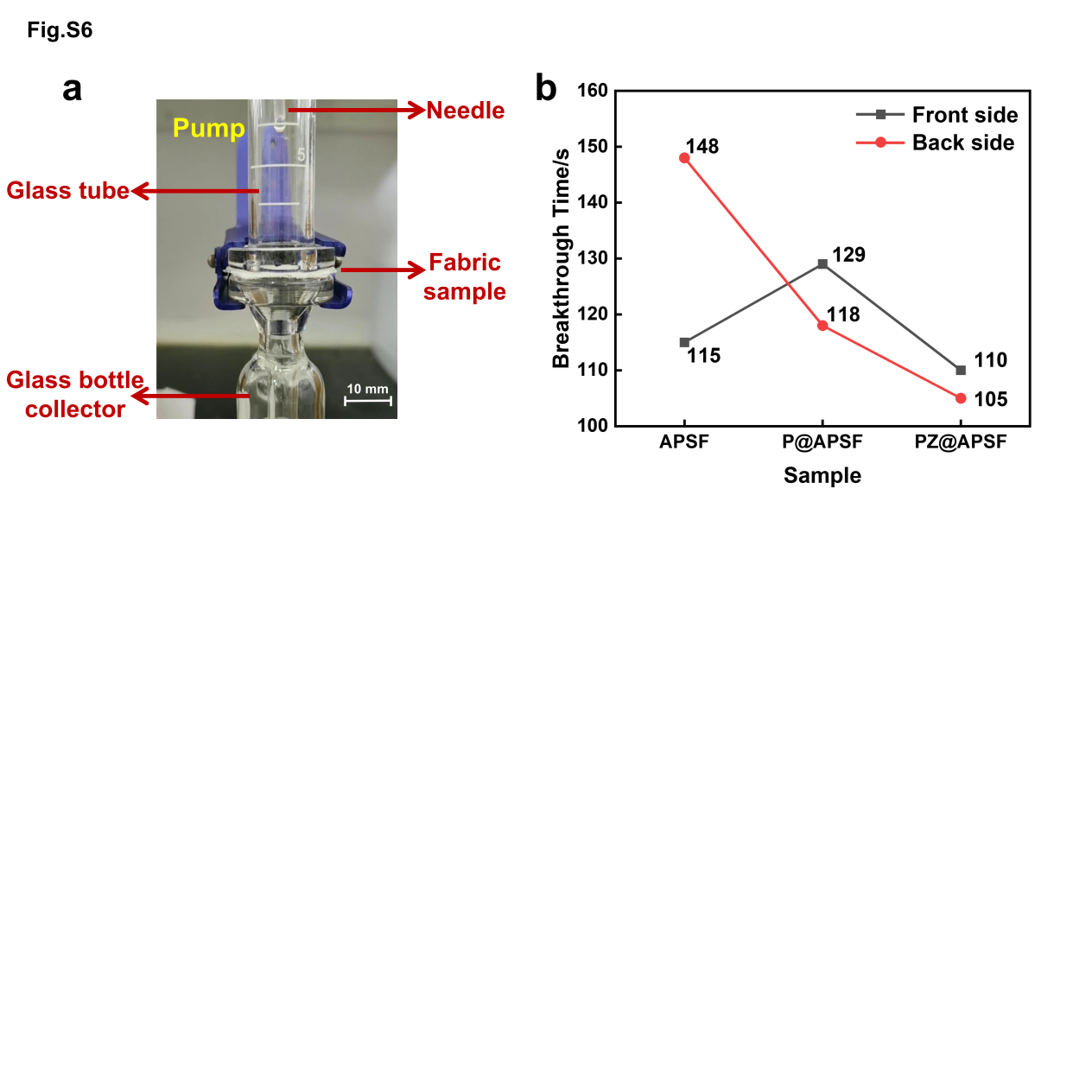


**Fig.S7.** a) Experimental set-up: a testing fabric was attached underneath a glass hollow cylinder, water was then pumped in, and breakthrough pressure was recorded as the pressure at which the water initiates passing through the fabric. b) Breakthrough time of both the front and back sides of the pristine polyester fabric, hydrophobic fabric treated with PDMS, and hydrophobic fabric treated with PDMS/ZnO.

**Fig.S8**





**Fig.S8.** Schematics of the elementary cells of a cross-section of an ideal yarn with a periodic hexagonal.

**Fig.S9**


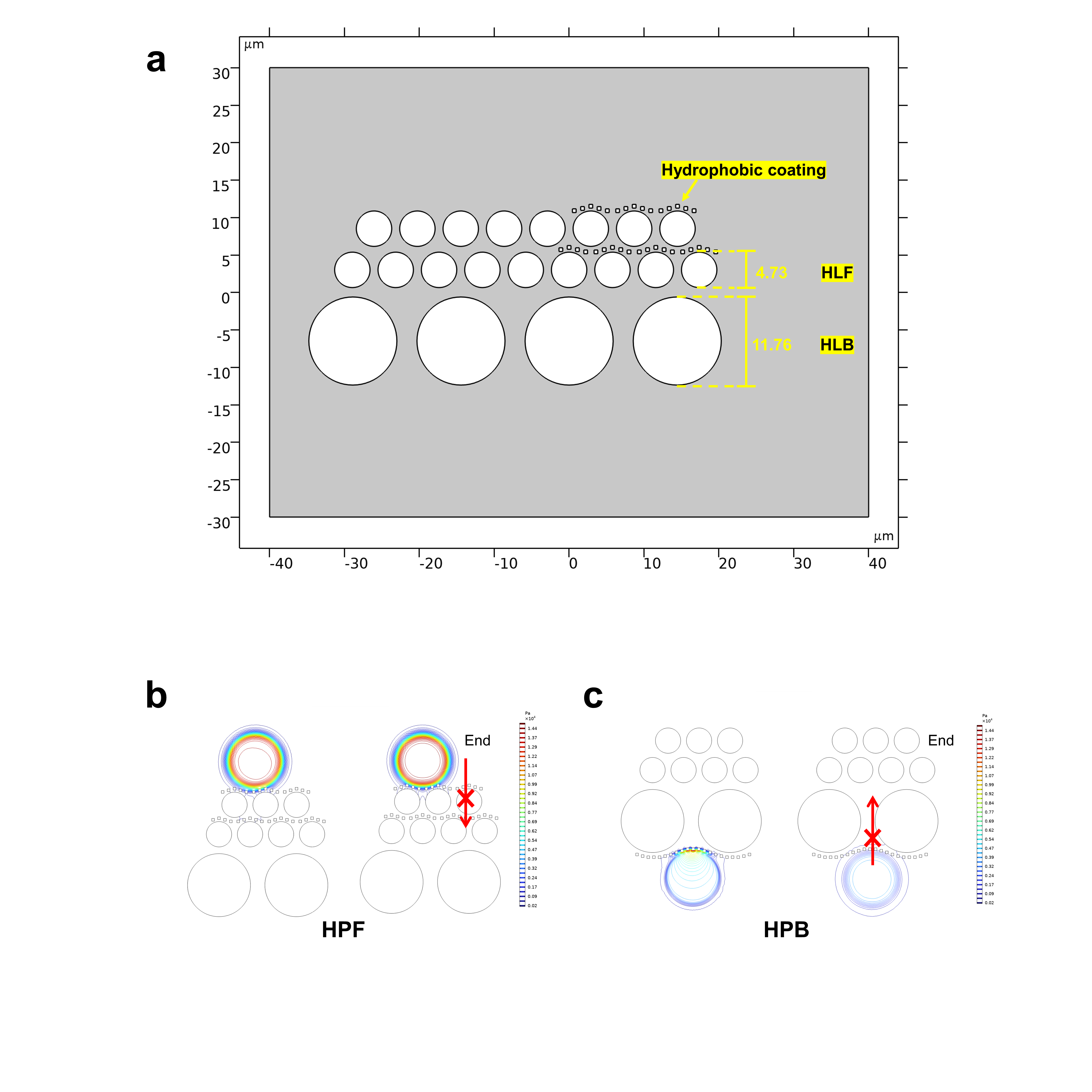


**Fig.S9.** a) Simplified cross-sectional schematic model of the hydrophobic finished fabric for fluid field simulation, including hydrophobic coating (small square), HLF layer (small circle) and HLB layer (large circle). b-c) Pressure distribution of water upon contact with the fabric from the HPF and HPB layers at a specific moment.

**Fig.S10**


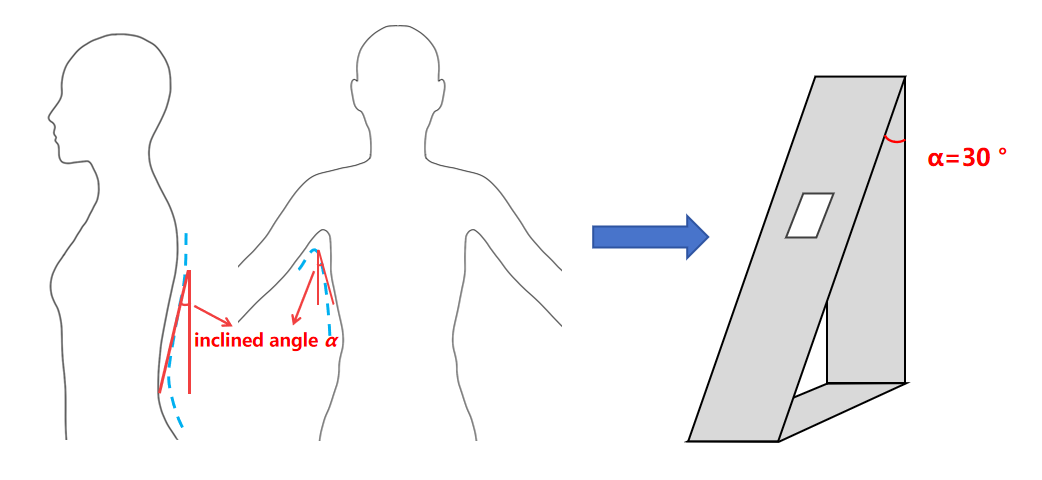


**Fig.S10.** Homemade setup consisting of a 30 ° inclined metal triangular frame.

**Table S1.** Chemical element contents of C, O, Si, and Zn on different fabric surfaces from EDS survey results.

| Sample | C/% | O/% | Si/% | Zn/% |
| --- | --- | --- | --- | --- |
| Pristine APSF | 61 | 39 | — | — |
| P@APSF | 42 | 30 | 28 | — |
| PZ3@APSF | 46 | 29 | 23 | 2 |

| **Table S2.** AOTI and transport time of various materials measured in the literature and this study. | | | |
| --- | --- | --- | --- |
| Sample | Material | AOTI/% | Transport time/s |
| Five-layer cross-laid fibrous webs^[1]^ | Polylactic acid–viscose | 165.60 | 1.5 |
| Knitting fabric^[2]^ | Polyester | 285.85 | ＜1.0 |
| Electrospinning membrane^[3]^ | Polypropylene/ bamboo fiber | 376.00 | 3.0 |
| Fabric with hydrophobic treatment^[4]^ | Silk | 526.16 | 8.3 |
| Fabric with electrospray treatment^[5]^ | Cotton | 800.00 | 15.0 |
| Woven fabric^[6]^ | Cotton/polycaprolactone | 1034.50 | 10.0 |
| Janus membrane^[7]^ | Polyester/nitrocellulose | 1246.00 | 6.0 |
| **This work** | **Polyester** | **499.57** | **＜1.0** |

**Supplementary Texts (S1-S2)**

**1. The capillary pressure at the transport channel.**

The liquid contact angle on both sides of the hydrophilic channel regarded as uniformly 0 ° in accordance with the results of the liquid management experiment (*θ_HLF_* = *θ_HLB_* = 0 °). As shown in Figure S8, approximating the internal structure of yarns as fibers with circular cross sections arranged in parallel yields a periodic hexagonal structure with three fibers packed to form an approximately triangular void cross-section. The circumference of this triangle is denoted as *L*, which accounts for half the circular circumference of the fiber cross-section. Each triangular pore can be seen as a capillary tube, with about the same number of triangular pores as fibers present. Therefore, by utilizing equations S1-3, one can calculate the equivalent radius *R* of the capillary. In this calculation, the capillary tube is simplistically considered to a circular shape to simplify the analysis.

| $S_{\Delta}=\sqrt{3}r^{2}-0.5\pi r^{2}$ | （1） |
| --- | --- |
| $R=\sqrt{\frac{S_{\Delta}}{\pi}}=0.226r$ | （2） |
| $2r=3568\times{10}^{-8}\sqrt{\frac{T}{\delta}}$ | （3） |

Where S_Δ_ refers to the area of the shaded area of the approximate-equilateral triangle, m^2^; *r* is the radius of the fiber, m; *R* is the equivalent radius of the capillary tube, m; *T* is the linear density of single fiber, tex; *δ* is the density of fiber, g*cm^-3^, and the fiber density of polyester is 1.38 g*cm^-3^. The calculation result of capillary pressure *ΔP* is as followed:

|  | T/tex | | r/*10^-6^m | R/*10^-6^ m | γ/N*m^-1^ | ΔP/Pa |
| --- | --- | --- | --- | --- | --- | --- |
|  | Yarn | Fiber |  |  |  |  |
| HLB | 5.55(36f) | 0.154 | 5.880 | 1.340 | 0.075 | 0.171＞0 |
| HLF | 9.33(384f) | 0.024 | 2.365 | 0.530 |  |  |

**2. The simulation of the fluid field of the fluid motion.**

Initially, the geometric structure of the hydrophobic finished textile was established, with the HLF layer represented by small circular cross-sections and the HLB layer by large circular cross-sections. Then, the hydrophobic coating, made of PDMS and ZnO, was delineated as squares distributed on the HLF layer. Following this, the two-dimensional spatial dimensions were selected in the model wizard to depict the dynamic change behavior of the water droplets. Subsequently, the two-phase flow and phase field module of COMSOL Multiphysics were utilized to simulate the fluid field during the fluid motion process. The “phase field” interface employed the Cahn-Hilliard equation, which includes a chemical potential, to delineate the diffusion interface separating the two phases. Moreover, the Navier-Stokes equation was employed to portray the momentum transfer and mass conservation. Material properties were then incorporated, boundary conditions were defined, following by the mesh was dissected and solved.

In the “phase-field” interface, the Cahn-Hilliard equation governs the two-phase fluid mechanics, tracking the diffusion interface of the insoluble two liquids. The diffusion interface is defined as the region of dimensionless phase field variables ranging from -1 to 1. When solved in COMSOL Multiphysics, the Cahn-Hilliard equation is divided into two equations^[8]^:

| $\frac{\partial\phi}{\partial t}+u\cdot\nabla\phi=\nabla\cdot\frac{\tau\lambda}{\varepsilon^{2}}\nabla\psi$ | （4） |
| --- | --- |
| $\psi=-\nabla\cdot\varepsilon^{2}\nabla\phi+\left( \phi^{2}-1 \right)\phi$ | （5） |

Where *u* is the fluid velocity (m/s), *τ* is the mobility (m^3^·s/kg), *λ* is the mixing energy density (N), *ε* is the interface thickness parameter. The *Φ* variable is called the phase field variable. The *ψ* variable is called the phase field auxiliary variable.

The multiphysical field coupling features define the density (*ρ_i_*) and viscosity (*μ_i_)* of the mixture varying smoothly across the biphase interface by the following expression^[9]^:

| $\rho_{i}=\rho_{2}+\left( \rho_{1}-\rho_{2} \right)V_{f1}$ | （6） |
| --- | --- |
| $\mu_{i}=\mu_{2}+\left( \mu_{1}-\mu_{2} \right)V_{f1}$ | （7） |
| $V_{f1}=\frac{1+\phi}{2}$ | （8） |
| $V_{f2}=\frac{1-\phi}{2}$ | （9） |

In the current model, air is defined as fluid 1, and water is defined as fluid 2.

The model incorporates mass and momentum transfer of incompressible fluid using the Navier-Stokes equation. To account for surface tension effects, the equation needs to include surface tension. Therefore, Navier-Stokes equation is provided as follows^[10]^:

| $\rho\frac{\partial u}{\partial t}+\rho\left( u\cdot\nabla\right)u=\nabla\cdot\left[ -pI+\mu\left( \nabla u+\left( \nabla u \right)^{T} \right) \right]+F_{\mathrm{st}}+\rho g$ | （10） |
| --- | --- |
| $\nabla\cdot u=0$ | （11） |
| $F_{\mathrm{st}}=G\nabla\phi$ |  |
| $G=\lambda\left[ -\nabla^{2}\phi+\frac{\phi\left( \phi^{2}-1 \right)}{\varepsilon^{2}} \right]=\frac{\lambda}{\varepsilon^{2}}\psi$ |  |

Where *ρ* is the fluid density (kg*m^-3^), *μ* is the fluid dynamic viscosity (Ns*m^-2^), *u* denotes the fluid velocity (m*s^-1^), *p* is the pressure of fluid (Pa), *g* is the gravity vector (m*s^-2^), *F_st_* is the surface tension acting at the air / water interface, *G* is the chemical potential (J*m^-3^).

**Supplementary References**

[1] Q. Zhen, H. Zhang, H. Li, J. Q. Cui, J. Cheng and Y. Liu, Acs Applied Polymer Materials, **2021**, 3, 3354.

[2] Z. J. Dong, Y. Q. Ding, H. L. Cong, C. Y. Chen and P. B. Ma, Text. Res. J., **2024**, 94, 36.

[3] N. Meng, Y. F. Zhang, Y. Y. Lin, C. C. Zhao, Z. L. Li, X. F. Wang, J. Y. Yu and B. Ding, Adv. Funct. Mater., **2023**, 33, 2305411.

[4] J. N. Pan, Z. F. Wang, M. X. Deng, J. Zhang, H. F. He, B. Wang, X. D. Liu and F. Y. Fu, J. Colloid Interface Sci., **2024**, 656, 587.

[5] H. X. Wang, H. Zhou, X. Wei, H. T. Niu and T. Lin, Advanced Materials Interfaces, **2018**, 5, 1800815.

[6] N. Mao, H. Peng, Z. Z. Quan, H. N. Zhang, D. Q. Wu, X. H. Qin, R. W. Wang and J. Y. Yu, ACS Appl. Mater. Interfaces, **2019**, 11, 44682.

[7] B. Dai, K. Li, L. X. Shi, X. Z. Wan, X. Liu, F. L. Zhang, L. Jiang and S. T. Wang, Adv. Mater., **2019**, 31, 1904113.

[8] H. A. A. Amiri and A. A. Hamouda, Int. J. Multiphase Flow, **2013**, 52, 22.

[9] D. Jacqmin, J. Comput. Phys., **1999**, 155, 96.

[10] V. E. Badalassi, H. D. Ceniceros and S. Banerjee, J. Comput. Phys., **2003**, 190, 371.
